# Enhancement of Anticancer Potential of Pterostilbene Derivative by Chalcone Hybridization

**DOI:** 10.3390/molecules26164840

**Published:** 2021-08-10

**Authors:** Kai-Wei Tang, Chien-Chih Ke, Chih-Hua Tseng, Yeh-Long Chen, Cherng-Chyi Tzeng, Yi-Jin Chen, Chia-Chi Hsu, Hsiao-Ting Tai, Ya-Ju Hsieh

**Affiliations:** 1 School of Pharmacy, College of Pharmacy, Kaohsiung Medical University, Kaohsiung 807, Taiwan; dadaking1107@gmail.com (K.-W.T.); chihhua@kmu.edu.tw (C.-H.T.); 2 Department of Medical Imaging and Radiological Sciences, College of Health Sciences, Kaohsiung Medical University, Kaohsiung 807, Taiwan; ccke@kmu.edu.tw (C.-C.K.); a133020@hotmail.com (Y.-J.C.); rty7535@yahoo.com.tw (C.-C.H.); 3 Drug Development and Value Creation Research Center, Kaohsiung Medical University, Kaohsiung 807, Taiwan; yeloch@kmu.edu.tw; 4 Department of Medical Research, Kaohsiung Medical University Hospital, Kaohsiung 807, Taiwan; 5 Department of Fragrance and Cosmetic Science, College of Pharmacy, Kaohsiung Medical University, Kaohsiung 807, Taiwan; 1010109@kmuh.org.tw; 6 Department of Pharmacy, Kaohsiung Municipal Ta-Tung Hospital, Kaohsiung 801, Taiwan; 7 Department of Medicinal and Applied Chemistry, College of Life Science, Kaohsiung Medical University, Kaohsiung 807, Taiwan; tzengch@kmu.edu.tw

**Keywords:** pterostilbene, chalcone hybridization, anticancer

## Abstract

Pterostilbene, a natural metabolite of resveratrol, has been indicated as a potent anticancer molecule. Recently, several pterostilbene derivatives have been reported to exhibit better anticancer activities than that of the parent pterostilbene molecule. In the present study, a series of pterostilbene derivatives were designed and synthesized by the hybridization of pterostilbene, chalcone, and cinnamic acid. The cytotoxic effect of these hybrid molecules was determined using two oral cancer cell lines, HSC-3 and OECM-1. (*E*)-3-(2-((*E*)-4-Hydroxystyryl)-4,6-dimethoxyphenyl)-1-(2-methoxyphenyl)prop-2-en-1-one (**4d**), with IC_50_ of 16.38 and 18.06 μM against OECM-1 and HSC-3, respectively, was selected for further anticancer mechanism studies. Results indicated that compound **4d** effectively inhibited cell proliferation and induced G2/M cell cycle arrest via modulating p21, cyclin B1, and cyclin A2. Compound **4d** ultimately induced cell apoptosis by reducing the expression of Bcl-2 and surviving. In addition, cleavage of PARP and caspase-3 were enhanced following the treatment of compound **4d** with increased dose. To conclude, a number of pterostilbene derivatives were discovered to possess potent anticancer potentials. Among them, compound **4d** was the most active, more active than the parent pterostilbene.

## 1. Introduction

Pterostilbene, a natural metabolite from dimethylation of resveratrol, is isolated from the *Pterocarpus santalinus* and is also found in blueberries and grapes [1]. The antioxidant activity of pterostilbene has been implicated in numerous potential preventive and therapeutic properties in neurological, carcinogenic, inflammation, metabolic, and bacterial disorders [2,3]. Similar to resveratrol, pterostilbene has been proved to be a potent anticancer agent [1] with anticancer activities through different mechanisms, such as modulation of signal transduction pathways [4], cell cycle regulation [5], cell differentiation [6], and oncogenes suppression [7]. Pterostilbene is generally safe for human use and the dose can be up to 250 mg/day in a clinical study [8]. However, more than five times the equivalent mean human intake dose of pterostilbene was required to significantly inhibit tumor growth in several in vivo studies [9,10,11,12].

The half-maximal inhibitory concentration (IC_50_) profile of pterostilbene in some cancer cell lines has been investigated, such as cervical cancer cell line (IC_50_ = 108.7 µM for HeLa; 44.45 µM for CaSki; 91.15 µM for SiHa) [13], human colon cell line (IC_50_ = 47.1 µM for HCT-116, 80.6 µM for HT-29) [14], breast cancer cell line (IC_50_ = 65 µM for MCF-7) [15], and cisplatin-resistance human oral cancer cell (IC_50_ = 98.29 µM for CAL27) [16]. Recently, several derivatives of pterostilbene were synthesized and reported with improved activity [17]. Hsieh et al. reported a series of ester derivatives from modification of pterostilbene. One of these derivatives, ANK-199 (**1**), exhibited potent activity against CAL27 and improved the IC_50_ from 98.29 to 32.6 µM, which showed a promising therapeutic potential [16,18]. Pterostilbene carboxaldehyde thiosemicarbazone (PTERC-T), a pterostilbene derivative, was reported to effectively decrease the IC_50_ of MCF-7 from 65 to 25 µM and reducing the tumor volumes in Ehrlich ascitic cell xenograft [15]. 3′-Hydroxypterostilbene (HPSB), a more potent pterostilbene analogue, effectively inhibited the growth of human colon cancer cells with IC_50_ = 40.2 µM for HCT-116 and 70.9 µM for HT-29 by inducing apoptosis and autophagy [14]. These results strongly suggest that the modification of pterostilbene may generate analogues with more enhanced anticancer activity. However, for therapeutic use, the efficacy of these analogues still needs to be improved.

Chalcone has the structure of 1,3-diaryl-2-propen-1-one, using three carbon units with α, β-unsaturated ketones to connect two sides of the aryl group. Chalcone is classified as a flavonoid and its derivatives can be widely found in natural fruits and vegetables. Chalcone derivatives also existed in many traditional therapeutical plants. Modern research of chalcone derivatives has revealed that its structure has diverse pharmacological effects, such as anticardiovascular and metabolic diseases [19,20], anticancer [21,22], antiretrovirus [23], anti-inflammation [24,25], and antibacteria activities [26]. In particular, the chalcone structure has the potential ability of autophagy-inducing, vascular-inhibition, and kinase-inhibition, which play an important role in anticancer drug development [27,28,29]. Cinnamic acid containing an aryl ring with α, β-unsaturated ketone, was reported to have anticancer activity. Therefore, both chalcone and cinnamic acid have been adapted as versatile structural moieties in many cases of cancer drug development [30].

**Figure 1:**
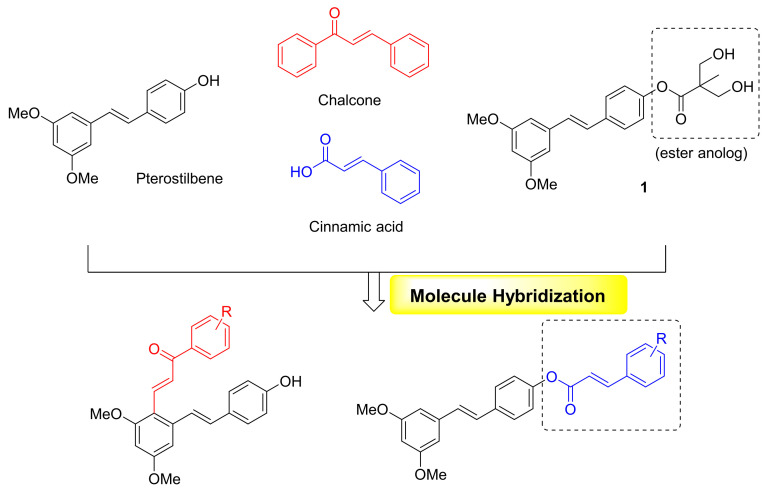
The design strategy of novel pterostilbene derivatives.

In a previous study, the combination of resveratrol and chalcone have been synthesized and tested for their anticancer activity. Resveratrol–chalcone derivatives were first published by Ruan et al. and found to be a potential antitubulin agent with IC_50_ values 6.07–350.20 µM [31]. Similar derivatives, published by Shin et al. in 2019, was found to against HCT-116 with IC_50_ values 3.42–91.41 µM [32]. In this study, pterostilbene, chalcone, and cinnamic acid were hybridized to generate two series of pterostilbene derivatives (Figure 1). Totally, 19 compounds were obtained and IC_50_ of each compound was tested on two human oral squamous carcinoma cell lines (OECM-1, HSC-3). According to the IC_50_ profile of all pterostilbene derivatives, (*E*)-3-(2-((*E*)-4-hydroxystyryl)-4,6-dimethoxyphenyl)-1-(2-methoxyphenyl)prop-2-en-1-one (**4d**), with the highest antiproliferation ability was selected for subsequent serial evaluations of anticancer performance as well as the mechanism.

## 2. Chemistry

**Scheme 1:**
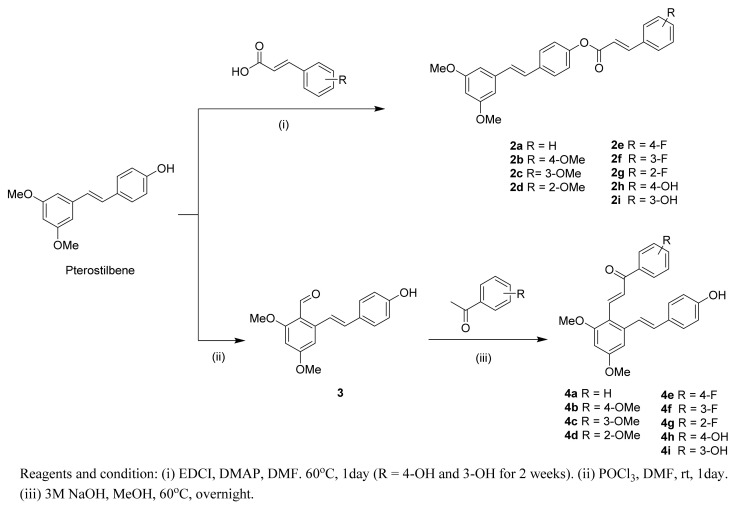
Synthesis of compound **2** and **4** series.

In the first series, pterostilbene was used as a starting material and the esterization was carried out with the selected cinnamic acid in the condition of EDCI and DAMP. 4-((*E*)-3,5-Dimethoxystyryl)phenyl cinnamate (**2a**) and its derivatives **2b**–**2g** were obtained in the yield of 60–80% (Scheme 1). However, the low yield of compound **2h** and **2i** (10–17%) may probably be due to the hydroxy group on the cinnamic acid which interferes with the esterification. In the second series, the Vilsmeier–Haak reaction of pterostilbene afforded *(E)*-2-(4-hydroxystyryl)-4,6-dimethoxybenzaldehyde (**3**) in 75% yield. Treatment of **3** with selected acetophenones gave (*E*)-3-(2-((*E*)-4-hydroxystyryl)-4,6-dimethoxyphenyl)-1-phenylprop-2-en-1-one (**4a**) and its derivatives **4b-4i** in the yield of 20–43% (Scheme 1).

**Scheme 2:**
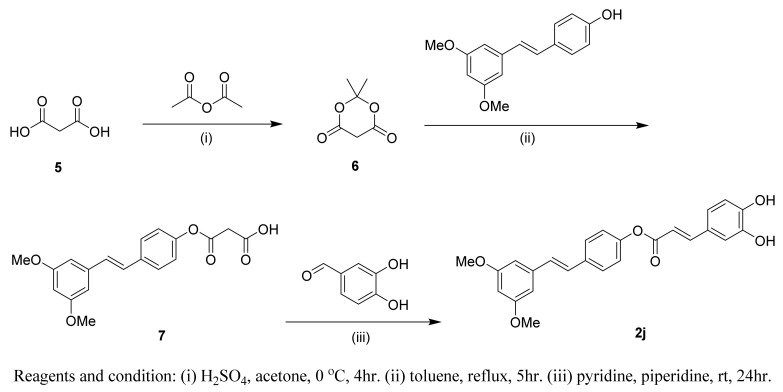
Synthesis of compound **2j**.

For compound **2j**, esterification of pterostilbene and caffeic acid gave complicated results, probably due to the interference of both hydroxyl groups in caffeic acid. We circumvented this dilemma by using malonic acid and acetic anhydride as started materials to obtain compound **6**. Then, pterostilbene was reacted with compound **6** and 3,4-dihydroxybenzaldehyde through two-step reactions to obtain compound **2j** (Scheme 2). All of the synthesized compounds were examined by NMR, LC-Mass for structural identifications, and the data were shown in the section of materials and methods (NMR spectra could be found in Supplementary Materials).

## 3. Biological Results and Discussion

### 3.1. Screening of Compound ***2*** and ***4*** Series by Cytotoxic Activity

In the beginning, the effect of each compound on the viability of two human oral squamous carcinoma cell (OSCC) lines was studied using MTT (dimethylthiazol-diphenyltetrazolium bromide) assay. Among these two cell lines, HSC-3 was one of the most highly aggressive OSCC line. The cell viability and IC_50_ was assessed after 72 h treatment with each synthesized compound at different concentrations (Table 1). Pterostilbene, with IC_50_ = 40.19 μM and > 50 μM in OECM-1 and HSC-3, respectively, was used as a positive control. Most of the pterostilbene–cinnamic acid derivatives **2a**–**2i** showed no cytotoxic effect against HSC-3 with IC_50_ of >50 μM with the exception of compound **2j**, which exhibited an IC_50_ of 18.53 μM. Compound **2g** exhibited comparable cytotoxicity to the positive pterostilbene against OECM-1. The IC_50_ of **2j** was 22.26 μM against OECM-1, while compounds **2a**–**2f**, **2h**, and **2i** were non-cytotoxic with IC_50_ of >50 μM, respectively.

Another series of pterostilbene-chalcone derivatives **4** exhibited more potent cytotoxic activity. Except for compound **4h** (IC_50_ > 40 μM), all the other tested compounds inhibited the viability of OECM-1 cells and the IC_50_ ranged from 9.62 to 22.8 μM. Among these compounds, compound **4i** exhibited the most potent cytotoxic activity against OECM-1 with IC_50_ 9.62 μM. Similar results were observed in HSC-3 cells; all tested compounds except compound **4h** (IC_50_ > 50 μM) showed antitumor activities. Among these compounds, the IC_50_ of **4c**–**d** and **4g** were less than 20 μM. Compound **4d** with IC_50_ 18.06 μM exhibited the most potent antitumor activity against HSC-3.

This evidence strongly supported our design, in that the cytotoxic effect of pterostilbene in OSCC lines could be enhanced apparently by a combination with chalcone. As a result, compound **4d**, one of the most potent anticancer compounds, was chosen for further mechanism studies.

### 3.2. Structure–Activity Relationship

**Figure 2:**
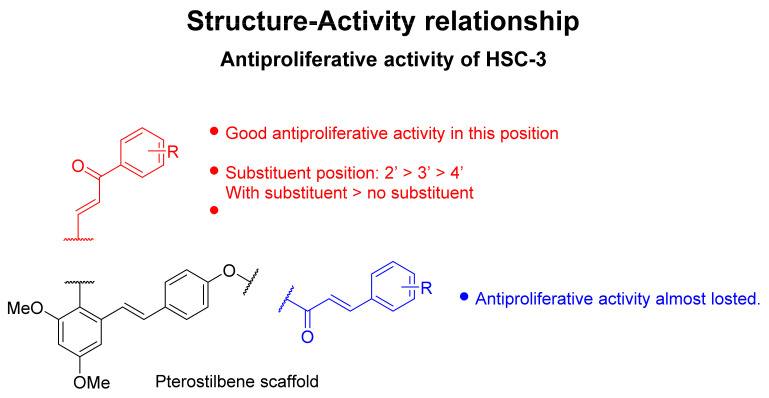
Structure–activity relationship between pterostilbene derivatives and HSC-3 antiproliferative activity.

From the results of preliminary screening, we figured out the structure–activity relationship (Figure 2). Compound **2** series, the ester derivatives of pterostilbene and selected cinnamic acid, was anticipated to show the characteristics of the prodrug, which could be metabolized to regenerate pterostilbene and cinnamic acid and exhibit synergistic anticancer activity. However, most of the compounds in this series showed no activity against OECM-1 and HSC-3. Only the caffeic acid derivative **2j**, 3′,4′-dihydroxy substituted, had the anticancer activity with IC_50_ 18.53 µM against HSC-3. We thus inferred that the anticancer activity was produced by caffeic acid moiety.

Compound **4** series were the derivatives of pterostilbene and chalcone. Except for compound **4h**, all compounds in this series exhibited more potent inhibitory activity on cell viability than pterostilbene. Moreover, the anticancer effects of compound **4** series with chalcone modification were better than compound **2** series. We also figured out the relationship between the substituent’s site and the anticancer activity in HSC-3. While comparing methoxy-substituted compounds (**4b**, **4c**, **4d**), the anticancer activities against HSC-3 in increasing order were as follows: **4b** (IC_50_: 28.93 µM) < **4c** (IC_50_: 18.22 µM) < **4d** (IC_50_: 18.06 µM). The result indicated that 2-substituted derivative was more active than the 3-substituted counterpart, which in turn was more active than the 4-substituted counterpart. The similar order was also found in fluoro-substituted compounds, **4e** (IC_50_: 19.13 μM) ≅ **4f** (IC_50_: 20.41 µM) < **4g** (IC_50_: 18.43 µM), and hydroxy-substituted compounds, **4h** (IC_50_ > 50 µM) < **4i** (IC_50_: 19.45 µM). There was no obvious difference on anticancer activity between different substituents in HSC-3. However, with substituent was more active than no substituent in most cases except for compound **4h**.

### 3.3. Compound ***4d*** Effectively Inhibited the Cell Proliferation through Modulation of Cell Cycle-Regulatory Proteins

**Figure 3:**
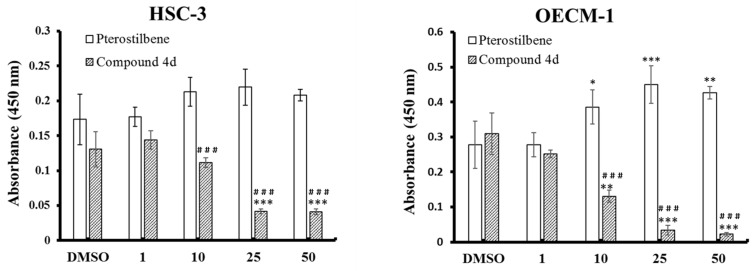
Inhibition of cell proliferation by compound **4d**. For cell proliferation analysis, HSC-3 and OECM-1 cells were treated by compound **4d** or pterostilbene with 0 (DMSO control), 1, 10, 25, and 50 μM for 24 h followed by BrdU incorporation assay. Incorporated BrdU in cells was determined by measurement of 450 nm absorbance using a spectrophotometer. The data is presented as the mean ± standard deviation of three independent experiments. Statistically significant differences of OD450 are indicated as follows: * *p* < 0.05, ** *p* < 0.01, *** *p* < 0.001 between each group and vehicle control; ^###^ *p* < 0.001 between compound **4d** treatment and the same concentration of pterostilbene treatment.

**Figure 4:**
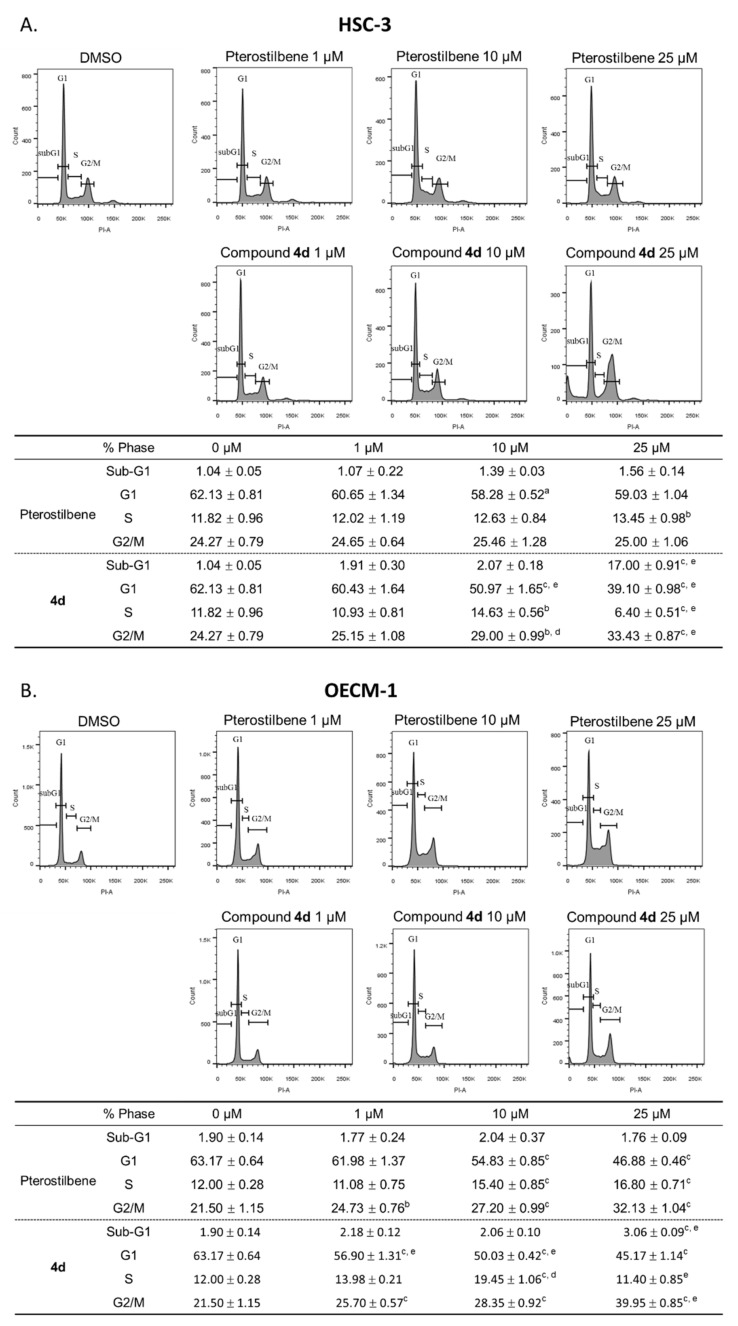
The effect of G2/M phase arrest induced by compound **4d**. For cell cycle analysis, (**A**) HSC-3 and (**B**) OECM-1 cells treated by compound **4d** or Pterostilbene for 24 h were subjected to PI staining, flow cytometric analysis, and quantitative analysis. Statistically significant differences are indicated as follows: ^a^ *p* < 0.05; ^b^ *p* < 0.01; ^c^ *p* < 0.001 between each group and vehicle control; ^d^ *p* < 0.01; ^e^ *p* < 0.001 between compound 4d treatment and the same concentration of pterostilbene treatment.

To further investigate the anticancer performance of compound **4d**, the cell proliferation and cell cycle of the two cell lines were evaluated after compound treatment. Cell proliferation of HSC-3 and OECM-1 cells was assayed by the incorporation of BrdU, an analogue of thymidine, which is taken up into the newly formed DNA by dividing cells. The result showed that the BrdU incorporation was significantly inhibited by compound **4d** at the concentrations of 10, 25, and 50 µM, while pterostilbene treatment did not inhibit but slightly enhanced the incorporation with the same dose (Figure 3). Specifically, as compared with the control group, treatment of 10, 25, and 50 µM of compound **4d** resulted in 15, 68, 70% decrease and 58, 89, 93% decrease in BrdU incorporation of HSC-3 and OECM-1, respectively (Figure 3). Cell cycle was analyzed in these cells after compound treatment. As shown in Figure 4A, G2/M arrest was induced remarkably in both cell lines after compound **4d** treatment. In HSC-3 cells, 29.0 and 33.4% of cells were induced arrested in the G2/M phase after 10 and 25 µM compound **4d** treatment, compared to 24.2% after DMSO treatment (Figure 4A). Treatment of 1, 10, and 25 µM of compound **4d** in OECM-1 cells induced 25.7, 28.3, and 40.0% of cells arresting in the G2/M phase, compared to 21.5% after DMSO treatment (Figure 4B). When compared with pterostilbene treatment, compound **4d** treatment induced significantly more cells arresting in the G2/M phase at higher concentrations (10 and 25 µM) in both lines, indicating the superior ability of compound **4d** to induce G2/M arrest of cancer cells over pterostilbene.

**Figure 5:**
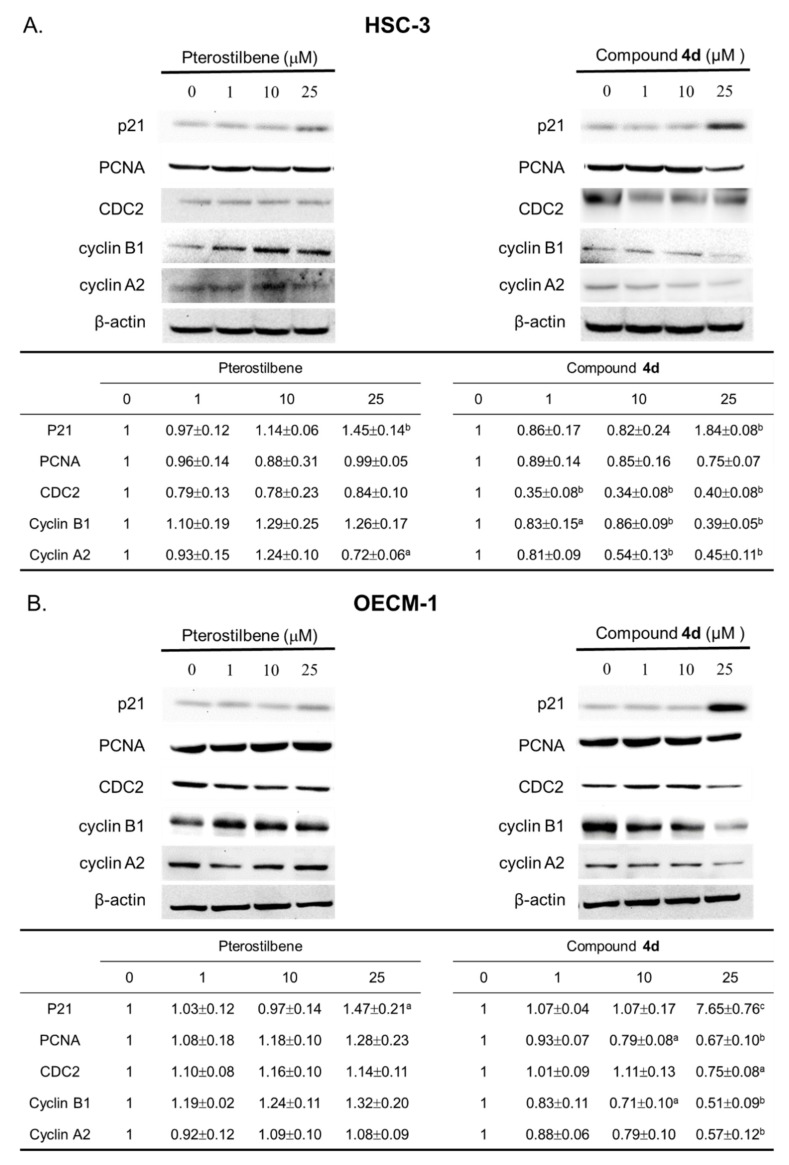
G2/M phase arrest induced by compound **4d** through modulation of cell cycle regulators. For protein expression analysis of cell cycle regulators, (**A**) HSC-3, and (**B**) OECM-1 cells were treated by compound **4d** or pterostilbene with 0 (DMSO control), 1, 10, and 25 μM. 24 h later, the cells were harvested, lysed, and the protein was isolated. Protein expression of p21, PCNA, cdc2, cyclin B1, cyclin A2, and β-actin was evaluated by Western blot using corresponding antibodies. The data were quantitative and indicated as follows: ^a^ *p* < 0.05; ^b^ *p* < 0.01; ^c^ *p* < 0.001 between each group and vehicle control.

Compound **4d** effectively inhibited cell proliferation and induced cell cycle arrest. The influence on the cell cycle regulatory molecules was further investigated. These include cdc2, cyclin-dependent kinases (CDKs), which work with cyclins to control the progression of the cell cycle, and p21, the CDK inhibitor known to suppress DNA synthesis and cell-cycle progression by inhibiting CDKs. Furthermore, proliferating cell nuclear antigen (PCNA), a cofactor of DNA polymerase-delta and essential for cell proliferation [33], was analyzed. The protein expression of these regulators was analyzed to elucidate the molecular mechanism. As shown in Figure 5, treatment of compound **4d** induced a remarkable increase of p21 and decrease of PCNA, cyclin B1, cyclin A2, and cdc2 expression in both HSC3 (Figure 5A) and OECM-1 (Figure 5B) cells. Negligible or no change in the expression of these proteins as observed in both lines after pterostilbene treatment. Together, these results indicated that compound **4d** effectively inhibited cell proliferation and induced cell-cycle arrest at the G2/M phase through modulation of cell cycle regulatory proteins in OECM-1 and HSC3 cells.

### 3.4. Compound ***4d*** Induced Apoptosis through Regulation of PARP and Caspase Activation

**Figure 6:**
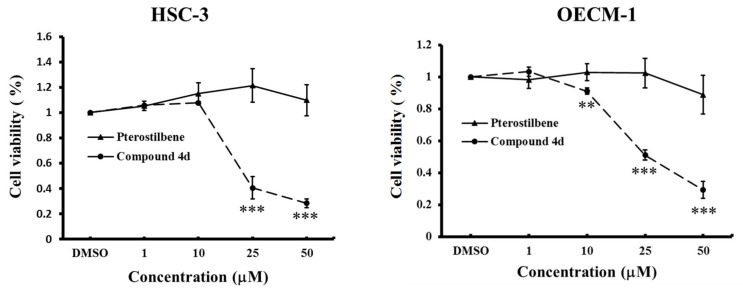
Cytotoxicity effect of pterostilbene and compound **4d.** HSC-3 and OECM-1 were treated by compound **4d** and pterostilbene with 0 (DMSO control), 1, 10, and 25 μM for 24 h. Cytotoxicity was measured by MTT assay. Statistically significant differences are indicated as follows: ** *p* < 0.01, *** *p* < 0.001 between each group and vehicle control.

**Figure 7:**
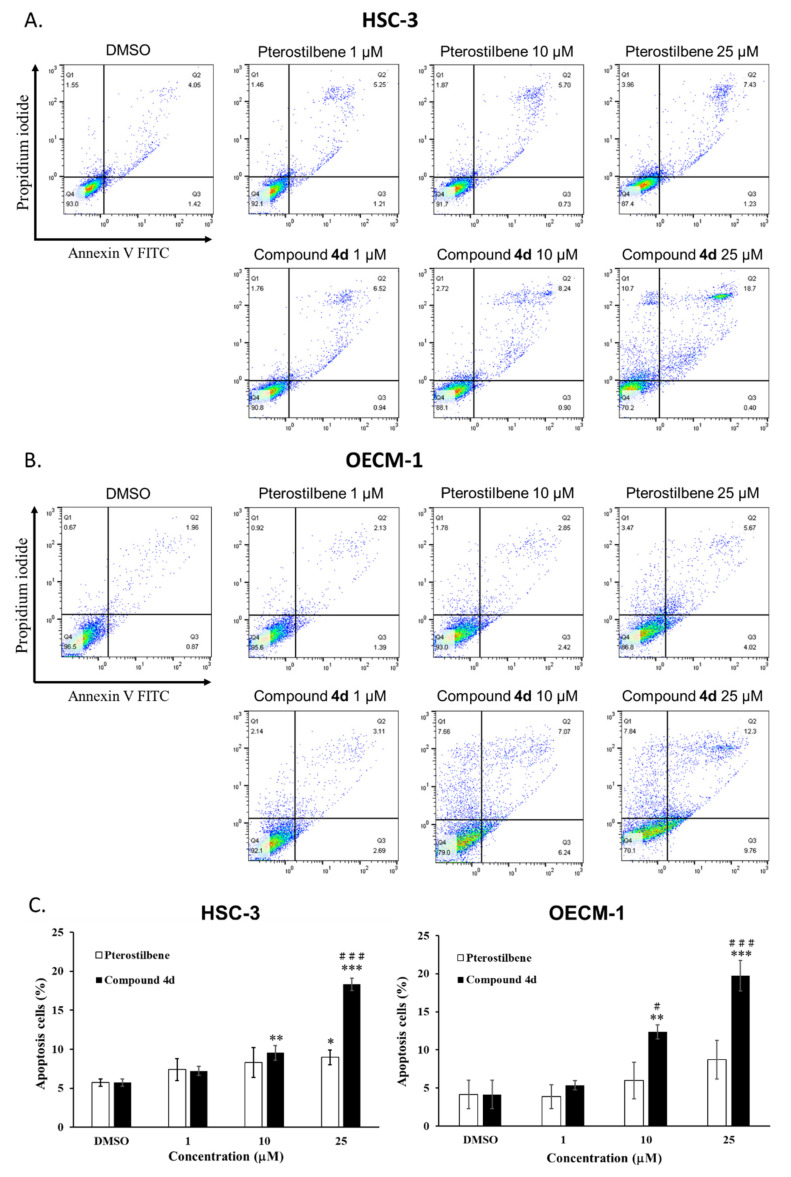
The effect of cell apoptosis induced by compound **4d**. (**A**) HSC-3 and (**B**) OECM-1 cells treated by compound **4d** or Pterostilbene for 24 h were subjected to annexin V and PI staining and flow cytometric analysis. (**C**) The quantitative analysis of (**A**,**B**). Statistically significant differences are indicated as follows: * *p* < 0.05, ** *p* < 0.01, *** *p* < 0.001 between each group and vehicle control; ^#^ *p* < 0.05, ^###^ *p* < 0.001 between compound 4d treatment and the same concentration of pterostilbene treatment.

**Figure 8:**
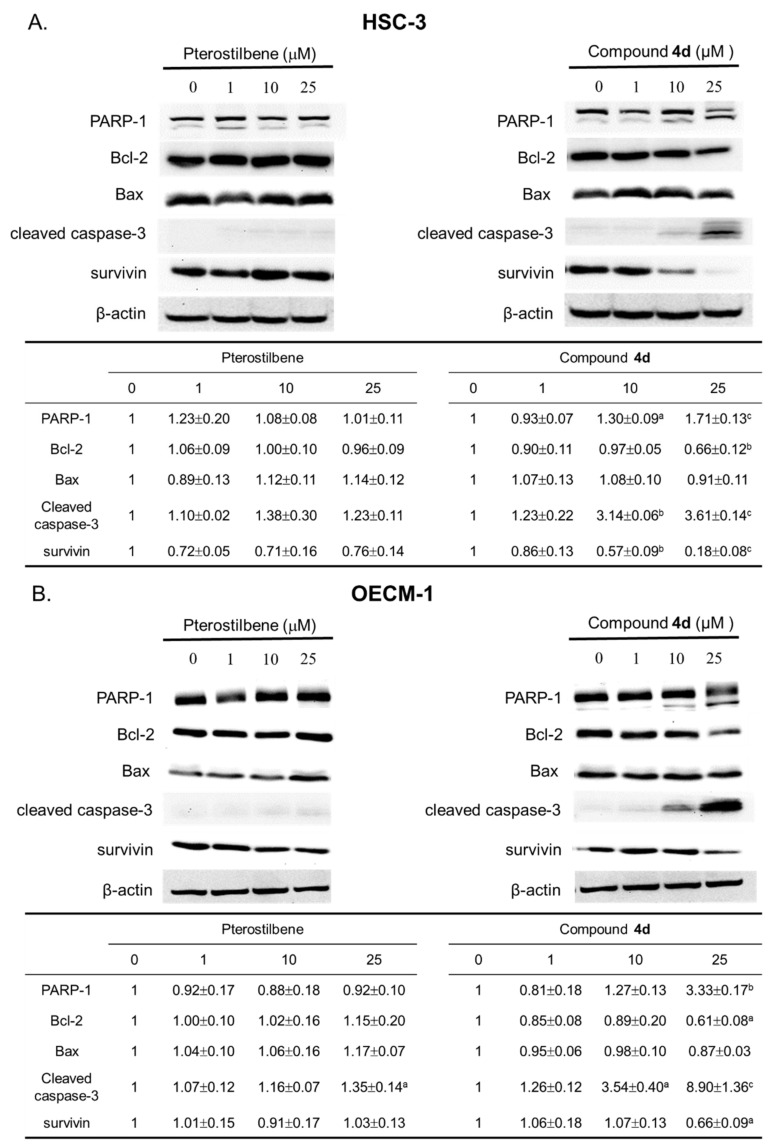
Cell apoptosis induced by compound **4d** through modulation of apoptosis-related proteins. For the analysis of apoptosis-associated protein expression, (**A**) HSC-3 and (**B**) OECM-1 cells were treated by compound **4d** or Pterostilbene with 0 (DMSO control), 1, 10, and 25 μM. 24 h later, the cells were harvested, lysed, and the protein was isolated. Protein expression of cleaved PARP, bcl-2, Bax, cleaved caspase 3, survivn and β-actin was evaluated by Western blot using corresponding antibodies. The data were quantitative and indicated as follows: ^a^ *p* < 0.05; ^b^ *p* < 0.01; ^c^ *p* < 0.001 between each group and vehicle control.

Compound **4d** has been indicated to induce proliferation inhibition and cell cycle arrest in OECM-1 and HSC-3 cells. Whether cell apoptosis, another crucial therapeutic and anticancer index, could be induced by compound **4d** was subsequently investigated. Cytotoxicity of compound **4d** to both lines was measured by MTT assay. The results showed that, with the treatment of 25 and 50 µM-compound **4d**, the cell viability dropped to 40 and 28% in HSC-3 and 51 and 29% in OECM-1 cells, as compared to 100% cell viability in control group (Figure 6). The IC_50_ of **4d** at 24, 48, and 72 h were 21, 18, and 18 µM on HSC3 cells, and were 25, 20, and16 µM on OECM-1 cells, respectively (Table 2). No influence on the viability of both cells was observed after treatment of pterostilbene, even with 50 µM. A similar result was observed by the evaluation of Annexin V staining and flow cytometry. As shown in Figure 7A,C, when treated with 1, 10, and 25 µM compound **4d**, apoptotic (Annexin V^+^) population of HSC-3 cells increased with treatment dose, from 7.2 ± 0.6, 9.5 ± 0.9, to 18.3 ± 0.8%, respectively, as compared to 5.3 ± 0.2% in the control (DMSO) group. For OECM-1 cells, apoptotic populations were 5.4 ± 0.6, 12.4 ± 0.9, and 19.7 ± 2.0% after treatment with 1, 10, and 25 µM compound **4d**, compared to 4.2 ± 1.9% in control group (Figure 7B,C). Note that the cell apoptosis in both lines was induced by pterostilbene treatment with a dose up to 25 µM (9.0 ± 0.9% and 8.7 ± 2.5% apoptotic population in HSC-3 and OECM-1 cells, respectively). To further elucidate the underneath mechanism, the expression of molecules involved in the apoptosis was evaluated. As shown in Figure 5B, the expression of antiapoptotic Bcl-2 decreased after cells were treated with 25 µM compound **4d**, while the pro-apoptotic Bax did not change in its expression. When a cell undergoes the final step of apoptosis, cleavage of Caspase 3 and PARP (Poly (ADP-ribose) Polymerase) are prerequisites. Our results showed that treatment of 10 and 25 µM compound **4d** remarkably increased the cleavage of PARP and caspase 3 in both lines (Figure 8A,B), as compared to those after pterostilbene treatment. In addition, the expression of survivin, which inhibits apoptosis protein family, was found decreased after treatment of 25 µM compound **4d**. Taken together, these results indicated compound **4d** was able to induce apoptosis through regulation of apoptotic related proteins.

## 4. Materials and Methods

### 4.1. General Information

Commercial reagents were used without additional purification. Melting points were tested with an Electrothermal IA9100 (Electrothermal, Staffordshire, UK) micro-melting point apparatus and were uncorrected. NMR spectra were performed with a Varian Unity 400 MHz spectrometer using tetramethylsilane (TMS, Merck, Darmstadt, Germany) as an internal standard and DMSO-*d_6_* as solvent. Chemical shifts were described as (ppm). Splitting patterns are presented as follows: s = singlet; d = doublet; t = triplet; q = quartet; dd = double doublet; m = multiplet. Analytical thin-layer chromatography (TLC) was carried out by an Art. 5554 Kieselgel 60 GF254 (Merck, Darmstadt, Germany) made by E. Merck. The spots of compounds were examined with a UV light indicator irradiated at 254 and 366 nm. Art. 7734 Kieselgel 60 GF254 (70–400 mesh, Merck, Darmstadt, Germany) produced by E. Merck was used for preparing column chromatography. Waters ZQ-4000 (Waters, Milford, MA, USA) liquid chromatography electrospray ionization mass spectrometry was used to record mass spectra.

### 4.2. General Procedure for the Synthesis of Compound ***2a–i***

Pterostilbene (0.42 g, 1.65 mmol), selected cinnamic acid (1.5 mmol), *N*-Ethyl-*N′*-(3-dimethylaminopropyl) carbodiimide hydrochloride (EDCI, 0.32 g, 1.65 mmol), and 4-dimethylaminopyridine (DMAP, 0.02 g, 0.15 mmol) were added together and dissolved in 20 mL acetonitrile. The mixture was refluxed at 60 °C for 24–48 h. After the reaction was completed (monitored by TLC), the mixture was concentrated in a vacuum. Then, the crude product was purified with silica gel column chromatography, using dichloromethane as the mobile phase. The target product was obtained in a 7–80% yield.

#### 4.2.1. 4-((*E*)-3,5-Dimethoxystyryl)phenyl Cinnamate (**2a**)

Compound **2a** was obtained in 63% yield as a white solid. Melting point: 137.3–138.2 °C. ^1^H NMR (400 MHz, DMSO-*d_6_*): δ 7.89 (d, *J* = 16 Hz, 1H, COCH), 7.83-7.81 (m, 2H, Ar-H), 7.69-7.66 (m, 2H, Ar-H), 7.48-7.47 (m, 3H, Ar-H), 7.32 (d, *J* = 16.4 Hz, 1H, CH_a_=CH), 7.25-7.23 (m, 2H, Ar-H), 7.19 (d, *J* = 16.4 Hz, CH=CH_b_), 6.90 (d, *J* = 16 Hz, 1H, ArCH), 6.81 (d, *J* = 2.4 Hz, 2H, Ar-H), 6.45-6.44 (m, 1H, Ar-H), 3.79 (s, 6H, OCH_3_). ^13^C NMR (100 MHz, DMSO-*d_6_*): δ 164.91, 160.67 (2C), 149.92, 146.50, 138.99, 134, 70, 133.84, 130.90, 128.99 (2C), 128.65 (2C), 127.98, 127.49, 122.11 (2C), 117.12, 104.51 (2C), 99.93, 55.19 (2C). MS (ESI): *m*/*z* [M + H]^+^ 387.13.

#### 4.2.2. 4-((*E*)-3,5-Dimethoxystyryl)phenyl (*E*)-3-(4-methoxyphenyl)acrylate (**2b**)

Compound **2b** was obtained in 69% yield as a white solid. Melting point: 149.2–150.3 °C. ^1^H NMR (400 MHz, DMSO-*d_6_*): δ 7.83 (d, *J* = 16 Hz, 1H, COCH), 7.79-7.76 (m, 2H, Ar-H), 7.67-7.65 (m, 2H, Ar-H), 7.31 (d, *J* = 16.4 Hz, 1H, CH_a_=CH), 7.22-7.15 (m, 3H, Ar-H, CH=CH_b_), 7.03-7.01 (m, 2H, Ar-H), 6.79 (d, *J* = 2.0 Hz, 2H, Ar-H), 6.73 (d, *J* = 16 Hz, 1H, ArCH), 6.43 (m, 1H, Ar-H), 3.82 (s, 3H, OCH_3_), 3.78 (s, 6H, OCH_3_). ^13^C NMR (100 MHz, DMSO-*d_6_*): δ 165.22, 161.54, 160.68 (2C), 150.04, 146.40, 139.03, 134.61, 130.59 (2C), 128.60, 128.03, 127.50 (2C), 126.50, 122.18 (2C), 114.50 (2C), 114.27, 104.51 (2C), 99.94, 55.40, 55.23 (2C). MS (ESI): *m*/*z* [M + H]^+^ 417.13.

#### 4.2.3. 4-((*E*)-3,5-Dimethoxystyryl)phenyl (*E*)-3-(3-methoxyphenyl)acrylate (**2c**)

Compound **2c** was obtained in 55% yield as a white solid. Melting point: 104.7–105.9 °C. ^1^H NMR (400 MHz, DMSO-*d_6_*): δ 7.85 (d, *J* = 16 Hz, 1H, COCH), 7.68-7.66 (m, 2H, Ar-H), 7.42-7.37 (m, 3H, Ar-H), 7.32 (d, *J* = 16 Hz, 1H, CH_a_=CH), 7.25-7.22 (m, 2H, Ar-H), 7.19 (d, *J* = 16 Hz, 1H, CH=CH_b)_, 7.06-7.03 (m, 1H, Ar-H), 6.94 (d, *J* = 16 Hz, 1H, ArCH), 6.80 (d, *J* = 2 Hz, 2H, Ar-H), 6.44-6.43(m, 1H, Ar-H), 3.82 (s, 3H, OCH_3_), 3.79 (s, 6H, OCH_3_). ^13^C NMR (100 MHz, DMSO-*d_6_*): δ 164.94, 160.67 (2C), 159.65, 149.92, 146.48, 139.00, 135.26, 134.73, 130.04, 128.65, 127.99, 127.52 (2C), 122.13 (2C), 121.31, 117.50, 117.03, 113.24, 104.51 (2C), 99.95, 55.28, 55.21 (2C). MS (ESI): *m*/*z* [M + H]^+^ 417.13.

#### 4.2.4. 4-((*E*)-3,5-Dimethoxystyryl)phenyl (*E*)-3-(2-methoxyphenyl)acrylate (**2d**)

Compound **2d** was obtained in 70% yield as a white solid. Melting point: 131.6–133.0 °C. ^1^H NMR (400 MHz, DMSO-*d_6_*): δ 8.08 (d, *J* = 16 Hz, 1H, COCH), 7.83-7.81 (m, 1H, Ar-H), 7.68-7.65 (m, 2H, Ar-H), 7.50-7.46 (m, 1H, Ar-H), 7.32 (d, *J* = 16.4 Hz, 1H, CH_a_=CH), 7.24-7.22 (m, 2H, Ar-H), 7.19 (d, *J* = 16.4 Hz, 1H, CH=CH_b_), 7.15-7.13 (m, 1H, Ar-H), 7.06-7.02 (m, 1H, Ar-H), 6.87 (d, *J* = 16.4 Hz, 1H, ArCH), 6.80 (d, *J* = 2.4 Hz, 2H, Ar-H), 6.44-6.43 (m, 1H, Ar-H), 3.90 (s, 3H, OCH_3_), 3.79 (s, 6H, OCH_3_). ^13^C NMR (100 MHz, DMSO-*d_6_*): δ 165.65, 160.67 (2C), 158.14, 149.97, 141.26, 139.01, 134.67, 132.61, 129.12, 128.62, 128.0, 127.49 (2C), 122.16 (2C), 122.0, 120.82, 117.13, 111.86, 104.50 (2C), 99.94, 55.72, 55.21 (2C). MS (ESI): *m*/*z* [M + H]^+^ 417.13.

#### 4.2.5. 4-((*E*)-3,5-Dimethoxystyryl)phenyl (*E*)-3-(4-fluorophenyl)acrylate (**2e**)

Compound **2e** was obtained in 83% yield as a white solid. Melting point: 146.6–147.9 °C. ^1^H NMR (400 MHz, DMSO-*d_6_*): δ 7.93-7.87 (m, 3H, Ar-H, COCH), 7.68-7.66 (m, 2H, Ar-H), 7.34-7.29 (m, 3H, Ar-H, CH_a_=CH), 7.24-7.22 (m, 2H, Ar-H), 7.19 (d, *J* = 16.8 Hz, 1H, CH=CH_b_), 6.88 (d, *J* = 16 Hz, 1H, ArCH), 6.80 (d, *J* = 2.0 Hz, 2H, Ar-H), 6.44-6.43 (m, 1H, Ar-H), 3.79 (s, 6H, OCH_3_). ^13^C NMR (100 MHz, DMSO-*d_6_*): δ 164.90, 163.56 (^1^*J*_CF_ = 247.9 Hz), 160.67 (2C), 149.92, 145.28, 139.0, 134.73, 131.10 (2C, ^3^*J*_CF_ = 9.1 Hz), 130.56 (^4^*J*_CF_ = 3 Hz), 128.66, 127.98, 127.51(2C), 122.13 (2C), 117.0, 116.05 (2C, ^2^*J*_CF_ = 21.2 Hz), 104.51, 99.94, 55.21 (2C). MS (ESI): *m*/*z* [M + H]^+^ 405.12.

#### 4.2.6. 4-((*E*)-3,5-Dimethoxystyryl)phenyl (*E*)-3-(3-fluorophenyl)acrylate (**2f**)

Compound **2f** was obtained in 76% yield as a white solid. Melting point: 119.7–120.9 °C. ^1^H NMR (400 MHz, DMSO-*d_6_*): δ 7.89 (d, *J* = 16 Hz, 1H, COCH), 7.77-7.74 (m, 1H, Ar-H), 7.69-7.66 (m, 3H, Ar-H), 7.54-7.49 (m, 1H, Ar-H), 7.34-7.30 (m, 2H, Ar-H, CH_a_=CH), 7.25-7.23 (m, 2H, Ar-H), 7.19 (d, *J* = 16.4 Hz, 1H, CH=CH_b_), 6.99 (d, *J* = 16 Hz, 1H, ArCH), 6.80 (d, *J* = 2.4 Hz, 2H, Ar-H), 6.44-6.43 (m, 1H, Ar-H), 3.79 (s, 6H, OCH_3_). ^13^C NMR (100 MHz, DMSO-*d_6_*): δ 164.72, 162.43 (^1^*J*_CF_ = 247.9 Hz), 160.66 (2C), 149.85, 145.08, 138.99, 136.37 (^3^*J*_CF_ = 7.6 Hz), 134.77, 130.95 (^3^*J*_CF_ = 8.3 Hz), 128.69, 127.96, 127.51 (2C), 125.16 (^4^*J*_CF_ = 2.3 Hz), 122.09 (2C), 118.79, 117.57 (^2^*J*_CF_ = 21.3 Hz), 114.76 (^2^*J*_CF_ = 22.0 Hz), 104.51 (2C), 99.94, 55.20 (2C). MS (ESI): *m*/*z* [M + H]^+^ 405.12.

#### 4.2.7. 4-((*E*)-3,5-Dimethoxystyryl)phenyl (*E*)-3-(2-fluorophenyl)acrylate (**2g**)

Compound **2g** was obtained in 74% yield as a white solid. Melting point: 98.2–106.6 °C. ^1^H NMR (400 MHz, DMSO-*d_6_*): δ 8.00-7.96 (m, 1H, Ar-H), 7.92 (d, *J* = 16 Hz, 1H, COCH), 7.69-7.67 (m, 2H, Ar-H), 7.58-7.52 (m, 1H, Ar-H), 7.38-7.30 (m, 3H, Ar-H, CH_a_=CH), 7.26-7.24 (m, 2H, Ar-H), 7.19 (d, *J* = 16.4 Hz, 1H, CH=CH_b_), 6.95 (d, *J* = 16.4 Hz, 1H, ArCH), 6.80 (d, *J* = 2.4 Hz, 2H, Ar-H), 6.44-6.43 (m, 1H, Ar-H), 3.79 (s, 6H, OCH_3_). ^13^C NMR (100 MHz, DMSO-*d_6_*): δ 164.65, 160.72 (^1^*J*_CF_ = 250.1 Hz), 160.66 (2C), 149.82, 138.98, 138.12 (^4^*J*_CF_ = 3.0 Hz), 134.80, 132.99 (^3^*J*_CF_ = 8.3 Hz), 129.60 (^4^*J*_CF_ = 2.3 Hz), 128.70, 127.95, 127.50 (2C), 125.12 (^3^*J*_CF_ = 3.1 Hz), 122.07 (2C), 121.45 (^2^*J*_CF_ = 10.6 Hz), 119.75 (^3^*J*_CF_ = 6.0 Hz), 116.17 (^2^*J*_CF_ = 22.0 Hz), 104.51 (2C), 99.95, 55.19 (2C). MS (ESI): *m*/*z* [M + H]^+^ 405.11.

#### 4.2.8. 4-((*E*)-3,5-Dimethoxystyryl)phenyl (*E*)-3-(4-hydroxyphenyl)acrylate (**2h**)

Compound **2h** was obtained in 10% yield as a yellow solid. Melting point: 196.4–198.3 °C. ^1^H NMR (400 MHz, DMSO-*d_6_*): δ 7.78 (d, *J* = 15.6 Hz, 1H, COCH), 7.67-7.65 (m, 4H, Ar-H), 7.31 (d, *J* = 16.4 Hz, CH_a_=CH), 7.22-7.16 (m, 3H, Ar-H, CH=CH_b_), 6.85-6.80 (m, 4H, Ar-H), 6.64 (d, *J* = 16 Hz, 1H, ArCH), 6.43 (m, 1H, Ar-H), 3.79 (s, 6H, OCH_3_). ^13^C NMR (100 MHz, DMSO-*d_6_*): δ 165.29, 160.67 (2C), 160.31, 150.07, 146.81, 131.02, 134.54, 130.76 (2C), 128.55, 128.02, 127.46 (2C), 124.94, 122.16 (2C), 115.87 (2C), 113.03, 104.49 (2C), 99.92, 55.21 (2C). MS (ESI): *m*/*z* [M + H]^+^ 403.10.

#### 4.2.9. 4-((*E*)-3,5-Dimethoxystyryl)phenyl (*E*)-3-(3-hydroxyphenyl)acrylate (**2i**)

Compound **2i** was obtained in 17% yield as a white solid. Melting point: 184.3–185.5 °C. ^1^H NMR (400 MHz, DMSO-*d_6_*) δ 9.71 (s, 1H, OH), 7.79 (d, *J* = 16 Hz, 1H, COCH), 7.68-7.66 (m, 2H, Ar-H), 7.34-7.14 (m, 7H, Ar-H, CH=CH), 6.91-6.89 (m, 1H, Ar-H), 6.82-6.75 (m, 3H, Ar-H, ArCH), 6.44 (m, 1H, Ar-H), 3.79 (s, 6H, OCH_3_). ^13^C NMR (100 MHz, DMSO-*d_6_*) δ 164.91, 160.67 (2C), 157.76, 149.94, 146.74, 139.01, 135.09, 134.69, 130.03, 128.65, 127.99, 127.50 (2C), 122.13 (2C), 119.59, 118.11, 116.88, 114.98, 104.52 (2C), 99.95, 55.21 (2C). MS (ESI): *m*/*z* [M + H]^+^ 403.10.

### 4.3. Procedure for the Synthesis of 4-((E)-3,5-Dimethoxystyryl)phenyl (E)-3-(3,4-dihydroxyphenyl) Acrylate (***2j***)

Malonic acid (4.20 g, 40.36 mmol), acetic anhydride (4.96 mL, 52.47 mmol), and H_2_SO_4_ (0.17 mL, 3.23 mmol) were mixed under ice bath. Then acetone (50 mL) was added to the mixture and reacted for 4 h. The mixture was put into −20 °C to recrystallize to get compound **5**. Compound **5** (0.51 g, 13 mmol) was mixed with pterostilbene (0.51 g, 2 mmol) and toluene (10mL) and refluxed 5 h. After reaction was completed (monitored by TLC), the mixture was concentrated in vacuum and purified with silica gel column chromatography (mobile phase dichloromethane:methanol = 100:1). Compound **6** was obtained. Compound **6** (0.51 g, 1.5 mmol) and 3,4-dihydroxybenzaldehye (0.22 g, 1.64 mmol) were stirred in pyridine (0.5 mL) and piperidine (0.05 mL) at room temperature for 1 day. After reaction was completed (monitored by TLC), the mixture was washed with dichloromethane and 1N HCl(aq) and purified with silica gel column chromatography (mobile phase dichloromethane:methanol = 50:1). Finally, white solid compound **2j** was available in 13% yeid. Melting point: 184.3–185.5 °C. ^1^H NMR (400 MHz, DMSO-*d_6_*) δ 7.72-7.65 (m, 3H, COCH, Ar-H), 7.31 (d, *J* = 16 Hz, 1H, CH_a_=CH), 7.21-7.10 (m, 5H, Ar-H, CH=CH_b_), 6.82-6.80 (m, 3H, Ar-H), 6.53-6.43 (m, 2H, Ar-H, ArCH), 3.79 (s, 6H, OCH_3_). ^13^C NMR (100 MHz, DMSO-*d_6_*) δ 165.29, 160.68 (2C), 155.10, 141.96, 147.26, 145.67, 139.04, 134.55, 128.57, 128.05, 127.48 (2C), 125.37, 122.20 (2C), 121.97, 115.80, 115.11, 112.81, 104.51 (2C), 99.94, 55.23 (2C). MS (ESI): *m*/*z* [M + H]^+^ 419.08.

### 4.4. Procedure for the Synthesis of (E)-2-(4-Hydroxystyryl)-4,6-dimethoxybenzaldehyde (***3***)

POCl_3_ (1 mL, 5.5 mmol) and dimethylformamide (DMF, 0.5 mL, 6 mmol) was mixed together at 0 °C for 30 min. Then pterostilbene (1.28 g, 5.00 mmol) was slowly added to the mixture and stirred in room temperature for 24 h. After the reaction was completed (monitored by TLC), the water was added into the mixture and put it overnight. The crude product was available after suction and dry in vacuum. 10 mL methanol was then used to wash the crude product. The yellow solid was obtained in 75% yield and pure enough to next synthesis. Melting point: 204.9.3–206.7 °C. ^1^H NMR (400 MHz, DMSO-*d_6_*): δ 10.41 (s, 1H, CHO), 9.69 (s, 1H, OH), 7.90 (d, 1H, *J* = 16 Hz, CH_a_=CH), 7.41-7.38 (m, 2H, Ar-H), 7.16 (d, 1H, *J* = 16 Hz, CH=CH_b_), 6.89-6.88 (m, 1H, Ar-H), 6.81-6.79 (m, 2H, Ar-H), 6.61-6.60 (m, 1H, Ar-H), 3.92 (s, 3H, OCH_3_), 3.90 (s, 3H, OCH_3_). ^13^C NMR (100 Hz, DMSO-*d_6_*): δ 189.79, 164.88, 164.51, 157.76, 142.05, 132.60, 128.29 (2C), 128.03, 123.29, 115.65 (2C), 115.06, 103.29, 97.23, 56.25, 55.75. MS (ESI): *m*/*z* [M + H]^+^ 285.

### 4.5. General Procedure for the Synthesis of Compound ***4a–i***

The selected acetophenone (2 mmol) was firstly dissolved in 10 mL methanol and 1 mL 3M NaOH_(aq.)_ at room temperature for 20 min. Then compound **3** (0.28 g, 1 mmol) was slowly added and refluxed 8 h. After the reaction was completed (monitored by TLC), the mixture was cooled to room temperature and concentrated in a vacuum. The crude product was then purified with silica gel column chromatography, using ethyl acetate:hexane = 1:2 as the mobile phase. The target product was concentrated in a vacuum and recrystallized in ethyl acetate and hexane. The pure compound was obtained in 17–45% yield.

#### 4.5.1. (*E*)-3-(2-((*E*)-4-Hydroxystyryl)-4,6-dimethoxyphenyl)-1-phenylprop-2-en-1-one (**4a**)

Compound **4a** was obtained in 40% yield as a yellow solid. Melting point: 158.1–160.4 °C. ^1^H NMR (400 MHz, DMSO-*d_6_*): δ 9.70(d, *J* = 0.4 Hz, 1H, OH), 8.05-7.95 (m, 3H, α-CH_a_=CH, Ar-H), 7.66-7.45 (m, 6H, α-CH=CH_b_, Ar-H), 7.36 (d, 1H, *J* = 16 Hz, β-CH_a_=CH), 7.04 1H, *J* = 16 Hz, β-CH=CH_b_), 6.82-6.79 (m, 3H, Ar-H), 6.62-6.61 (m, 1H, Ar-H), 3.93 (s, 3H, OCH_3_), 3.89 (s, 3H, OCH_3_). ^13^C NMR (100 Hz, DMSO-*d*_6_): δ 189.77, 161.68, 160.76, 157.73, 141.73, 138.11, 132.73, 132.49, 128.77 (2C), 128.29, 128.21, 128.06, 127.87, 123.90, 123.58, 115.63 (2C), 114.45, 104.12, 97.66, 55.98, 55.51. MS (ESI): *m*/*z* [M + H]^+^ 387.16.

#### 4.5.2. (*E*)-3-(2-((*E*)-4-Hydroxystyryl)-4,6-dimethoxyphenyl)-1-(4-methoxyphenyl)prop-2-en-1-one (**4b**)

Compound **4b** was obtained in 39% yield as a yellow solid. Melting point: 172.3–174.3 °C. ^1^H NMR (400 MHz, DMSO-*d*_6_): δ 9.70 (s, 1H, OH), 8.00-7.94 (m, 3H, α-CH_a_=CH, Ar-H), 7.59 (d, 1H, *J* = 15.6 Hz, α-CH=CH_b_), 7.47-7.44 (m, 2H, Ar-H), 7.30 (d, 1H, *J* = 16.4 Hz, β-CH_a_=CH), 7.06-7.02 (m, 3H, β-CH=CH_b_, Ar-H), 6.82-6.79 (m, 3H, Ar-H), 6.61 (d, 1H, *J* = 2.0 Hz, Ar-H), 3.92 (s, 3H, OCH_3_), 3.89 (s, 3H, OCH_3_), 3.84 (s, 3H, OCH_3_). ^13^C NMR (100 Hz, DMSO-*d*_6_): δ 187.96, 162.94, 161.48, 160.61, 157.72, 141.46, 137.20, 132.33, 130.85, 130.41 (2C), 128.20 (2C), 127.93, 124.03, 123.69, 115.66 (2C), 114.70, 114.03 (2C), 104.00, 97.69, 55.97, 55.51 (2C). MS (ESI): *m*/*z* [M + H]^+^ 417.18.

#### 4.5.3. (*E*)-3-(2-((*E*)-4-Hydroxystyryl)-4,6-dimethoxyphenyl)-1-(3-methoxyphenyl)prop-2-en-1-one (**4c**)

Compound **4c** was obtained in 44% yield as a yellow solid. Melting point: 201.3–203.1 °C. ^1^H NMR (400 MHz, DMSO-*d*_6_): δ 9.67 (s, 1H, OH), 8.03 (d, 1H, *J* = 16 Hz, α-CH_a_=CH), 7.57-7.52 (m, 2H, Ar-H, α-CH=CH_b_), 7.47-7.40 (m, 4H, Ar-H), 7.30 (d, 1H, *J* = 16 Hz, β-CH_a_=CH), 7.21-7.19 (m, 1H, Ar-H), 7.04 (d, 1H, *J* = 16 Hz, β-CH=CH_b_, Ar-H), 6.82-6.78 (m, 3H, Ar-H), 6.61 (d, 1H, *J* = 2.4 Hz, Ar-H), 3.92 (s, 3H, OCH_3_), 3.89 (s, 3H, OCH_3_), 3.77 (s, 3H, OCH_3_). ^13^C NMR (100 Hz, DMSO-*d*_6_): δ 189.43, 161.68, 160.73, 159.46, 157.71, 141.68, 139.58, 138.16, 132.45, 129.89, 128.17 (2C), 127.85, 123.92, 123.59, 120.49, 118.82, 115.62 (2C), 114.46, 112.44, 104.15, 97.65, 55.98, 55.50, 55.16. MS (ESI): *m*/*z* [M + H]^+^ 417.17.

#### 4.5.4. (*E*)-3-(2-((*E*)-4-Hydroxystyryl)-4,6-dimethoxyphenyl)-1-(2-methoxyphenyl)prop-2-en-1-one (**4d**)

Compound **4d** was obtained in 43% yield as a yellow solid. Melting point: 170.2–171.9 °C. ^1^H NMR (400 MHz, DMSO-*d*_6_): δ 9.69 (s, 1H, OH), 8.03 (d, 1H, *J* = 16 Hz, α-CH_a_=CH), 7.52-7.48 (m, 2H, Ar-H), 7.38-7.35 (m, 2H, Ar-H), 7.28 (d, 1H, *J* = 16 Hz, α-CH=CH_b_), 7.16-7.12 (m, 2H, Ar-H, β-CH_a_=CH), 7.06-7.00 (m, 2H, Ar-H, β-CH=CH_b_), 6.80-6.78 (m, 3H, Ar-H), 6.58 (d, 1H, *J* = 2.0 Hz, Ar-H), 3.88 (s, 3H, OCH_3_), 3.87 (s, 3H, OCH_3_), 3.70 (s, 3H, OCH_3_). ^13^C NMR (100 Hz, DMSO-*d*_6_): δ 192.77, 161.54, 160.59, 157.70, 157.44, 141.24, 137.36, 132.55, 132.26, 129.44, 129.10, 129.03, 128.14 (2C), 127.87, 123.33, 120.52, 115.59 (2C), 114.34, 112.21, 103.95, 97.67, 55.87, 55.52, 55.48. MS (ESI): *m*/*z* [M + H]^+^ 417.19.

#### 4.5.5. (*E*)-1-(4-Fluorophenyl)-3-(2-((*E*)-4-hydroxystyryl)-4,6-dimethoxyphenyl)prop-2-en-1-one (**4e**)

Compound **4e** was obtained in 29% yield as a yellow solid. Melting point: 199.6–201.2 °C. ^1^H NMR (400 MHz, DMSO-d6): δ 9.70 (s, 1H, OH), 8.06-8.00 (m, 3H, Ar-H, α-CH_a_=CH), 7.60 (d, 1H, *J* = 15.6 Hz, α-CH=CH_b_), 7.47-7.45 (m, 2H, Ar-H), 7.35-7.29 (m, 3H, Ar-H, β-CH_a_=CH), 7.04 (d, 1H, J = 16 Hz, β-CH=CH_b_), 6.81-6.79 (m, 3H, Ar-H), 6.61 (m, 1H, Ar-H), 3.93 (s, 3H, OCH_3_), 3.89 (s, 3H, OCH_3_). ^13^C NMR (100 Hz, DMSO-d6): δ 188.35, 164.74 (^1^*J*_CF_ = 249.4 Hz), 161.73, 160.82, 157.75, 141.84, 138.29, 134.76 (^4^*J*_CF_ = 3.0 Hz), 132.61, 131.00 (2C, ^2^*J*_CF_ = 9.1 Hz), 130.40, 128.22 (2C), 127.87, 123.48 (2C, ^3^*J*_CF_ = 8.3 Hz), 115.88, 115.66 (2C), 114.38, 104.09, 97.67, 56.00, 55.53. MS (ESI): *m*/*z* [M + H]^+^ 405.15.

#### 4.5.6. (*E*)-1-(3-Fluorophenyl)-3-(2-((*E*)-4-hydroxystyryl)-4,6-dimethoxyphenyl)prop-2-en-1-one (**4f**)

Compound **4f** was obtained in 20% yield as a yellow solid. Melting point: 189.5–191.6 °C. ^1^H NMR (400 MHz, DMSO-*d*_6_): δ 9.70 (s, 1H, OH), 8.05 (d, 1H, *J* = 15.2 Hz, α-CH_a_=CH), 7.81-7.79 (m, 1H, Ar-H), 7.70-7.68 (m, 1H, Ar-H), 7.58-7.45 (m, 5H, Ar-H, α-CH=CH_b_), 7.31 (d, 1H, *J* = 16 Hz, β-CH_a_=CH), 7.04 (d, 1H, *J* = 16 Hz, β-CH=CH_b_), 6.82-6.79 (m, 3H, Ar-H), 6.61 (m, 1H, Ar-H), 3.93 (s, 3H, OCH_3_), 3.89 (s, 3H, OCH_3_). ^13^C NMR (100 Hz, DMSO-*d*_6_): δ 188.69, 162.26 (^1^*J*_CF_ = 244.1 Hz), 161.89, 160.92, 157.75, 141.98, 140.49 (^4^*J*_CF_ = 6.0 Hz), 138.86, 132.67, 130.98 (^3^*J*_CF_ = 8.3 Hz), 128.22 (2C), 127.85, 124.27, 123.43 (2C), 119.6 (^2^*J*_CF_ = 21.3 Hz), 115.65 (2C), 114.43 (^2^*J*_CF_ = 24.2 Hz), 104.21, 97.66, 56.03, 55.55. MS (ESI): *m*/*z* [M + H]^+^ 405.15.

#### 4.5.7. (*E*)-1-(2-Fluorophenyl)-3-(2-((*E*)-4-hydroxystyryl)-4,6-dimethoxyphenyl)prop-2-en-1-one (**4g**)

Compound **4g** was obtained in 23% yield as a yellow solid. Melting point: 164.8–166.2 °C. ^1^H NMR (400 MHz, DMSO-*d*_6_): δ 9.68 (s, 1H, OH), 7.91 (dd, 1H, *J* = 2.0, 16.0 Hz, α-CH_a_=CH), 7.75-7.70 (m, 1H, Ar-H), 7.65-7.59 (m, 1H, Ar-H), 7.41-7.39 (m, 2H, Ar-H), 7.35-7.29 (m, 3H, Ar-H, α-CH=CH_b_), 7.21 (d, 1H, *J* = 16 Hz, β-CH_a_=CH), 7.01 (d, 1H, *J* = 16 Hz, β-CH=CH_b_), 6.82-6.79 (m, 3H, Ar-H), 6.60 (d, 1H, *J* = 2.0 Hz, Ar-H), 3.90 (s, 3H, OCH_3_), 3.88 (s, 3H, OCH_3_). ^13^C NMR (100 Hz, DMSO-*d*_6_): δ 189.45 (^3^*J*_CF_ = 2.3 Hz), 161.99, 160.93, 160.00 (^1^*J*_CF_ = 248.5 Hz), 157.76, 141.95, 139.19, 133.77 (^2^*J*_CF_ = 8.8 Hz), 132.84, 130.35 (^4^*J*_CF_ = 2.7 Hz), 128.21 (2C), 127.74, 127.33 (^3^*J*_CF_ = 3.1 Hz), 127.17, 124.20 (^3^*J*_CF_ = 3.4 Hz), 122.94, 116.50 (^2^*J*_CF_ = 22.5 Hz), 115.60 (2C), 113.87, 104.18, 97.70, 55.95, 55.54. MS (ESI): *m*/*z* [M + H]^+^ 405.15.

#### 4.5.8. (*E*)-1-(4-Hydroxyphenyl)-3-(2-((*E*)-4-hydroxystyryl)-4,6-dimethoxyphenyl)prop-2-en-1-one (**4h**)

Compound **4h** was obtained in 17% yield as a yellow solid. Melting point: 234.4 °C (decomposed). ^1^H NMR (400 MHz, DMSO-*d*_6_): δ 7.96 (d, 1H, *J* = 16 Hz, α-CH_a_=CH), 7.88-7.85 (m, 2H, Ar-H), 7.57 (d, 1H, *J* = 16 Hz, α-CH=CH_b_), 7.47-7.45 (m, 2H, Ar-H), 7.30 (d, 1H, *J* = 16 Hz, β-CH_a_=CH), 7.04 (d, 1H, *J* = 16 Hz, β-CH=CH_b_), 6.86-6.79 (m, 5H, Ar-H), 6.60 (d, 1H, *J* = 2.0 Hz, Ar-H), 3.92 (s, 3H, OCH_3_), 3.88 (s, 3H, OCH_3_). ^13^C NMR (100 Hz, DMSO-*d*_6_): δ 187.70, 161.94, 161.40, 160.54, 157.71, 141.33, 136.74, 132.21, 130.68 (2C), 129.51, 128.19 (2C), 127.96, 124.21, 123.79, 115.68 (2C), 115.43 (2C), 114.83, 104.01, 97.70, 56.00, 55.53. MS (ESI): *m*/*z* [M + H]^+^ 403.15.

#### 4.5.9. (*E*)-1-(3-Hydroxyphenyl)-3-(2-((*E*)-4-hydroxystyryl)-4,6-dimethoxyphenyl)prop-2-en-1-one (**4i**)

Compound **4i** was obtained in 37% yield as a yellow solid. Melting point: 207.3 °C (decomposed). ^1^H NMR (400 MHz, DMSO-*d*_6_): δ 9.81 (s, 1H, OH), 9.68 (s, 1H, OH), 8.00 (d, 1H, *J* = 15.6 Hz, α-CH_a_=CH), 7.56 (d, 1H, *J* = 15.6 Hz, α-CH=CH_b_), 7.47-7.45 (m, 2H, Ar-H), 7.39-7.28 (m, 4H, Ar-H, β-CH_a_=CH), 7.06-7.01 (m, 2H, Ar-H, β-CH=CH_b_), 6.83-6.79 (m, 3H, Ar-H), 6.61 (d, 1H, *J* = 2.4 Hz, Ar-H), 3.93 (s, 3H, OCH_3_), 3.89 (s, 3H, OCH_3_). ^13^C NMR (100 Hz, DMSO-*d*_6_): δ 189.63, 161.63, 160.76, 157.72, 157.69, 141.71, 139.54, 137.86, 132.53, 129.81, 128.20 (2C), 127.85, 123.92, 123.40, 119.86, 118.96, 115.64 (2C), 114.43, 114.30, 104.77, 97.70, 55.98, 55.50. MS (ESI): *m*/*z* [M + H]^+^ 403.15.

### 4.6. Biological Studies

#### 4.6.1. Cell Viability Assays

All human oral squamous carcinoma cancer cell lines HSC-3 and OECM-1 were cultured in MEM, RPMI-1640 medium (Gibco, Grand Island, NY, USA), respectively. Medium were supplemented with 10% fetal bovine serum (FBS), 1% non-essential amino acids (NEAA) (Gibco, Grand Island, NY, USA), 0.11 g/L sodium pyruvate (SP) (Applichem, Panreac, Spain), and 1% penicillin/ streptomycin (P/S) (BioInd, Israel) in 5% CO_2_ at 37 °C. Cell viability was measured by the 3-(4,5-dimethylthiazol-2-yl)-2,5-diphenyltetrazolium bromide (MTT) assay. HSC-3 and OECM-1 cells (5 × 10^3^ cells per well) were seeded in 96 well-plate and incubated with test compounds for fixed time intervals. Then, the medium was discarded and rinsed by PBS. Afterward, cells were added with 100 μL fresh serum-free containing 10% MTT solution (Cyrusbioscience, Taipei, Taiwan). After incubation at 37 °C for 1 h, MTT-containing solution was removed and the formazan crystals were solubilized with 120 μL of DMSO to detect activities of succinate dehydrogenase in living cells. The absorption levels of each well was measured at 590 nm by a microplate spectrophotometer (Multiskan GO Microplate Spectrophotometer, Thermo Scientific, Vantaa, Finland).

#### 4.6.2. BrdU Incorporation Assay

HSC-3 and OECM-1 cells (5 × 10^3^ cells per well) were seeded in 96 well-plate and incubated with test compounds for 24 h. The effect of test agents on cell proliferation was analyzed using a cell proliferation ELISA kit (Millipore Corp., Bedford, MA, USA) with the incorporation of 5-bromo-20-deoxyuridine (BrdU) for 2 h. The absorbance of BrdU uptake was quantified using an ELISA reader at 450 nm (Multiskan GO Microplate Spectrophotometer, Thermo Scientific, Vantaa, Finland).

#### 4.6.3. Apoptosis Detection by Annexin V Staining and Flow Cytometry

HSC-3 and OECM-1 cell death induced by test compounds was accessed by using an Annexin V/PI staining kit (BioLegend, San Diego, CA) following the manual protocol. Briefly, cells were seeded at a density of 5 × 10^5^ cells per 60 mm Petri dish. After treatment with different doses of test drugs for 24 h, cells were detached by trypsin and washed by Cell Staining Buffer. Cells were then stained with the Annexin V-FITC and PI (5 μg/mL) in the dark at room temperature for 15 min. The number of apoptosis cells was analyzed by flow cytometry using a BD LSR II Cell Analyzer (BD Biosciences, San Jose, CA, USA). Data were collected at rates of 70–300 events per second at the lowest or medium flow rate, with 10,000 events being recorded per sample. The proportion of apoptotic cells was evaluated by FlowJo (FlowJo, Ashland, OR, USA).

#### 4.6.4. Cell Cycle Analysis by Flow Cytometry

HSC-3 and OECM-1 cells (5 × 10^5^) were cultured and incubated with test compounds at different concentrations for 24 h. Cells were harvested using accutase (BioLegend, San Diego, CA, USA) and washed twice with PBS. Next, cells were fixed by 70% ethanol at 4 °C overnight. The fixed cells were washed by PBS and suspended with 500 μL of mixture containing PI (40 μg/mL), RNase A (50 μg/mL). Follow by a 30-min incubation in the dark at room temperature, cell pellets were analyzed by flow cytometry BD LSR II (BD Biosciences, San Jose, CA, USA). Cell cycle profiles were measured at rates of 70–300 events per second at the lowest or medium flow rate from 10,000 events per sample. The percentages of cells in different cell cycle phases were evaluated by FlowJo (FlowJo, Ashland, OR, USA).

#### 4.6.5. Western Blot Assay

HSC-3 and OECM-1 cells were treated with DMSO or test agents for 24 h. Whole-cell lysates were washed twice by PBS and lysed in RIPA buffer, containing 1% protease inhibitor cocktail and phosphatase inhibitor. Protein samples were separated by sodium dodecyl sulfate-polyacrylamide gel electrophoresis (SDS-PAGE) and transferred to polyvinylidene difluoride membranes. After blocking with nonfat milk for 1h at room temperature, membranes were hybridized with primary antibodies at 4 °C overnight. The blot was then incubated with horseradish peroxidase-conjugated secondary antibody for 1 h at room temperature. Protein expression signals were detected using the Multi-function Gel Image System, MultiGel-21(TOPBIO). Bands of β-actin served as a loading control. The following antibodies p21(#2947), PCNA (#13110), CDC2 (#9116), cyclin B1 (#12231), cyclin A2 (#4656), PARP (#9542), Bcl-2 (#15071), cleaved Caspase-3 (#9664) and survivin (#2808) were purchased from Cell Signaling Technology (Beverly, MA, USA). BAX (SC-7480) was obtained from Santa Cruz Biotechnology (Santa Cruz, CA, USA) and β-actin was bought from BioLegend (San Diego, CA, USA).

#### 4.6.6. Statistical Analysis

All experimental data were repeated at least three times and were presented as the mean ± standard deviation (SD). Comparison of data among different concentrations of pterostilbene, compound **4d**, and/or vehicle was analyzed by ANOVA followed by Bonferroni post hoc tests.

## 5. Conclusions

**Figure 9:**
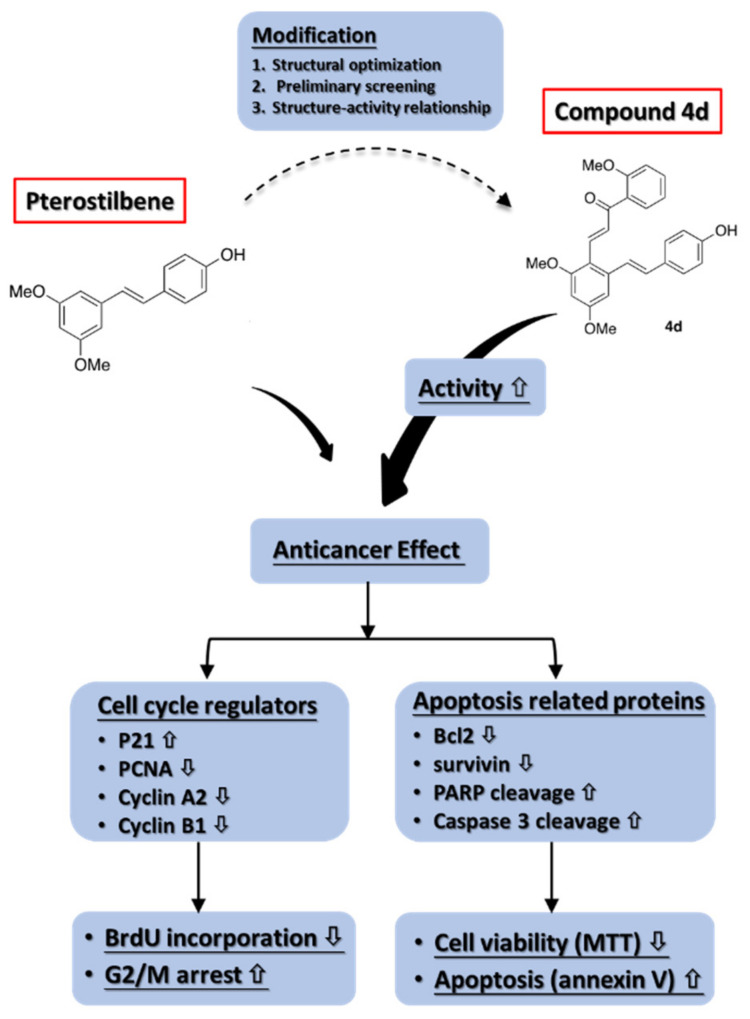
Schematic representation of the mechanism of compound **4d** in anticancer activity.

In the present work, two series of pterostilbene derivatives were synthesized and evaluated in vitro for their anticancer activities (Figure 9). Among them, compound **4d** showed the most potent activity against the cell growth of HSC-3 and OECM-1 with IC_50_ values of 16.83 and 18.06 μM, respectively, and therefore, was selected as a new lead for further comprehensive evaluation and mechanism investigation. Results indicated that compound **4d** effectively inhibited cell proliferation, induced G2/M cell cycle arrest via modulation of the p21, cyclin B1, and cyclin A2. Compound **4d** ultimately induced cell apoptosis by the decrease of antiapoptotic protein Bcl-2 and surviving. In addition, cleavage of PARP and caspase-3 were induced and increased following the treatment of compound **4d** with increased dose. It is noteworthy that the anticancer effects above were observed in cells after the treatment of compound **4d** with a dose range 10~50 μM, while pterostilbene did not show any effect with the same dose, suggesting a superior anticancer potential of compound **4d** over its parent compound, pterostilbene.

## Figures and Tables

**Table 1:** In vitro IC_50_ values (µM) of compound **2**, **4** series in OSCC lines.

Compound		IC_50_ (μM) of MTT Assay after 72 h	
No.	R	OECM-1	HSC-3
**2a**	H	>50	>50
**2b**	4-OMe	>50	>50
**2c**	3-OMe	>50	>50
**2d**	2-OMe	>50	>50
**2e**	4-F	>50	>50
**2f**	3-F	>50	>50
**2g**	2-F	40.24 ± 0.06	>50
**2h**	4-OH	>50	>50
**2i**	3-OH	>50	>50
**2j**	3,4-diOH	22.26 ± 0.24	18.53 ± 0.43
**4a**	H	22.80 ± 0.27	43.44 ± 0.75
**4b**	4-OMe	11.08 ± 0.05	28.93 ± 0.09
**4c**	3-OMe	11.50 ± 0.01	18.22 ± 0.11
**4d**	2-OMe	16.38 ± 0.10	18.06 ± 0.05
**4e**	4-F	13.70 ± 0.01	19.13 ± 0.26
**4f**	3-F	16.39 ± 0.03	20.41 ± 0.41
**4g**	2-F	17.34 ± 0.15	18.43 ± 0.09
**4h**	4-OH	46.21 ± 2.01	>50
**4i**	3-OH	9.62 ± 0.34	19.45 ± 0.11
Pterostilbene		40.19 ± 0.69	>50

All data were presented by mean ± standard deviation (*n* = 3).

**Table 2:** In vitro IC_50_ values (µM) of compound **4** at 24, 48, and 72 h after treatment.

IC_50_ (µM) of Compound 4d		
Time (h)	HSC-3	OECM-1
24	21.72 ± 0.55	25.07 ± 0.67
48	17.98 ± 0.41 ^a^	20.47 ± 0.55 ^a^
72	18.06 ± 0.05 ^a^	16.38 ± 0.10 ^a^

All data were presented by mean ± standard deviation (*n* = 3). Statistically significant differences are indicated as ^a^ *p* < 0.001 in groups compared with 24 h treatment.

## Data Availability

Not applicable. All data which was showed in this study was originally made by the authors.

## Supplementary Materials

The following are available, NMR spectra of each tested compound.

## Sample Availability

Samples of the compounds **2a–j** and **4a–i** are available from the authors.
