# Supplementary material for: Enhancement of Anticancer Potential of Pterostilbene Derivative by Chalcone Hybridization
Source: Molecules. 2021 Aug 10;26(16):4840. doi: 10.3390/molecules26164840 (PMC8401670; doi:10.3390/molecules26164840)

Supplementary Information

Enhancement of anticancer potential of pterostilbene derivative by chalcone hybridization

Kai-Wei Tang [1, †], Chien-Chih Ke [2, 3, †], Chih-Hua Tseng [1, 3, 4, 5, 6], Yeh-Long Chen [3, 7], Cherng-Chyi Tzeng [7], Yi-Jin Chen [2], Chia-Chi Hsu [2], Hsiao-Ting Tai [2] and Ya-Ju Hsieh [2, 3, 8, *]

1. School of Pharmacy, College of Pharmacy, Kaohsiung Medical University, Kaohsiung 807, Taiwan; dadaking1107@gmail.com
2. Department of Medical Imaging and Radiological Sciences, College of Health Sciences, Kaohsiung Medical University, Kaohsiung 807, Taiwan; ccke@kmu.edu.tw
3. Drug Development and Value Creation Research Center, Kaohsiung Medical University, Kaohsiung 807, Taiwan; chihhua@kmu.edu.tw
4. Department of Fragrance and Cosmetic Science, College of Pharmacy, Kaohsiung Medical University, Kaohsiung 807, Taiwan
5. Department of Medical Research, Kaohsiung Medical University Hospital, Kaohsiung 807, Taiwan
6. Department of Pharmacy, Kaohsiung Municipal Ta-Tung Hospital, Kaohsiung 801, Taiwan
7. Department of Medicinal and Applied Chemistry, College of Life Science, Kaohsiung Medical University, Kaohsiung 807, Taiwan; yeloch@kmu.edu.tw; tzengch@kmu.edu.tw
8. Department of Medical Research, Kaohsiung Medical University Hospital, Kaohsiung, Taiwan; 1010109@kmuh.org.tw

* Correspondence: yjhsieh@kmu.edu.tw; Tel.: +886-7-3121101 (ext. 2356)

† These authors contributed equally to this work.

NMR spectra of each tested compound (The integration of one proton was set to 10)

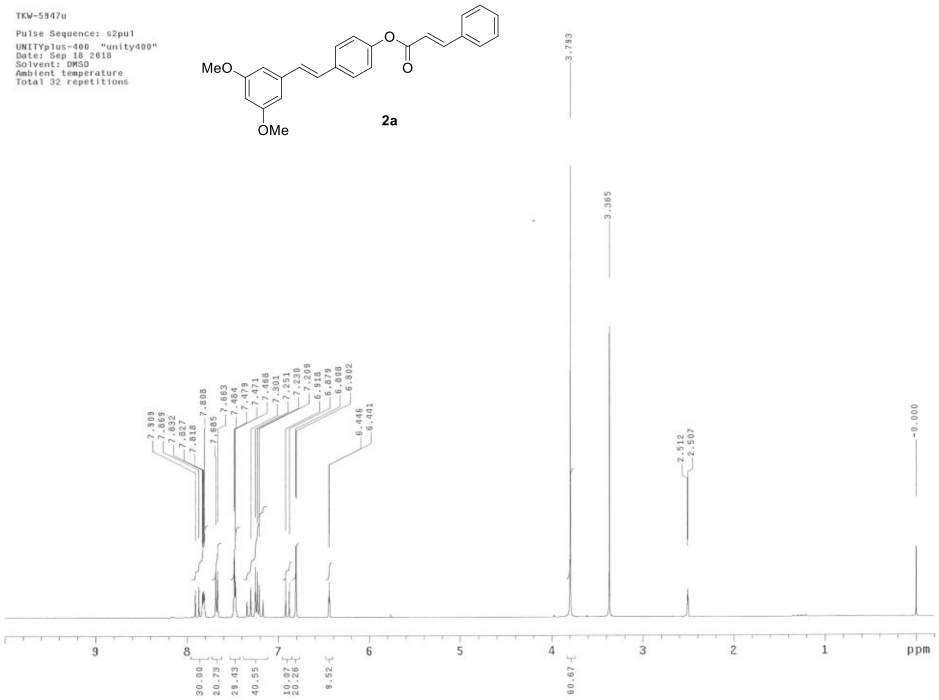

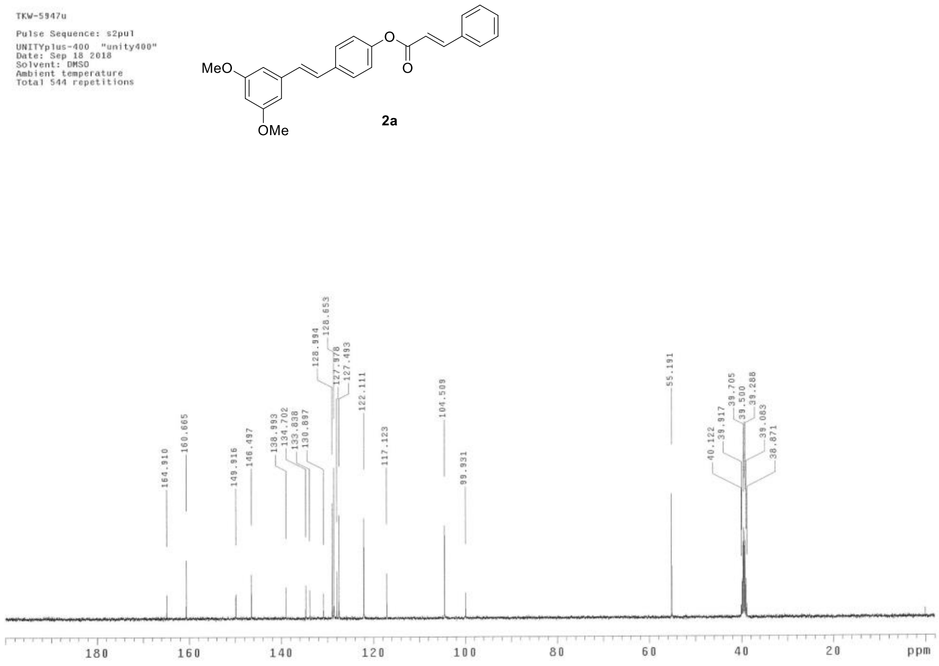

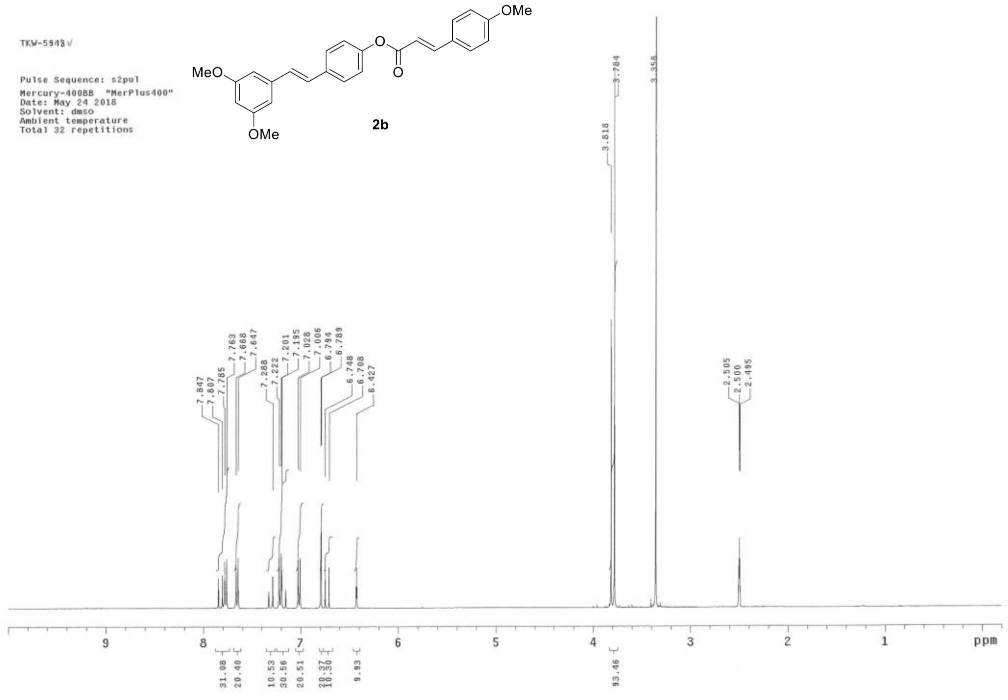
TKW-594B
Pulse Sequence: s2pul
Mercury-400BB "MerPlus400"
Date: May 24 2018
Solvent: dmso
Ambient temperature
Total 32 repetitions
MeO
OMe
OMe
O
O
2b
3.818
3.784
3.358
2.505
2.500
2.495
ppm

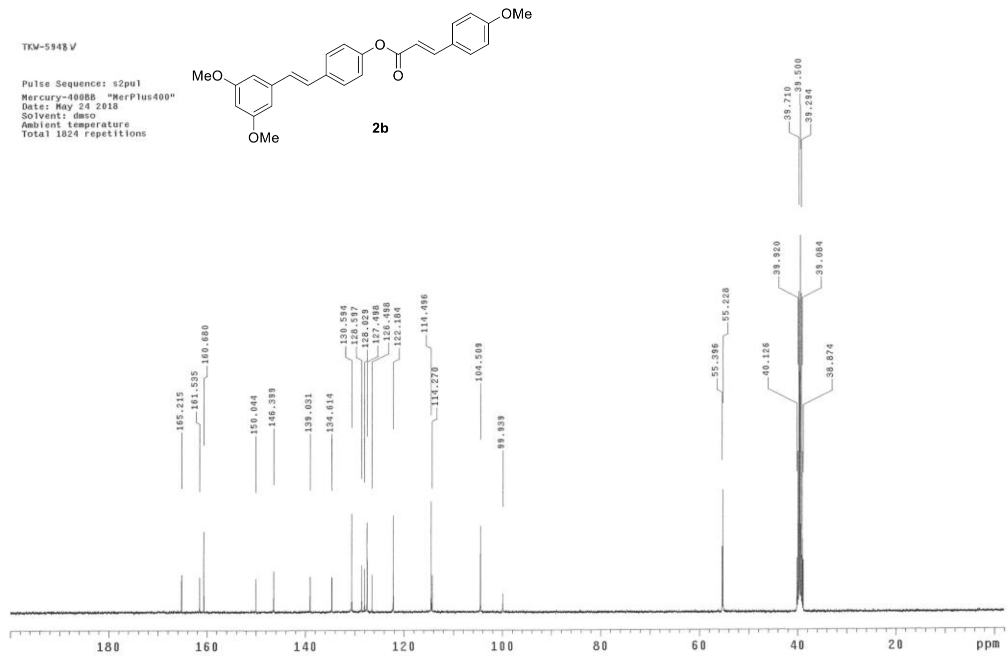
TKW-594B
Pulse Sequence: s2pul
Mercury-400BB "MerPlus400"
Date: May 24 2018
Solvent: dmso
Ambient temperature
Total 1824 repetitions
MeO
OMe
OMe
O
O
2b
165.215
161.535
160.680
150.044
146.399
139.031
134.614
114.496
114.270
104.509
99.939
55.396
55.228
40.126
38.874
ppm

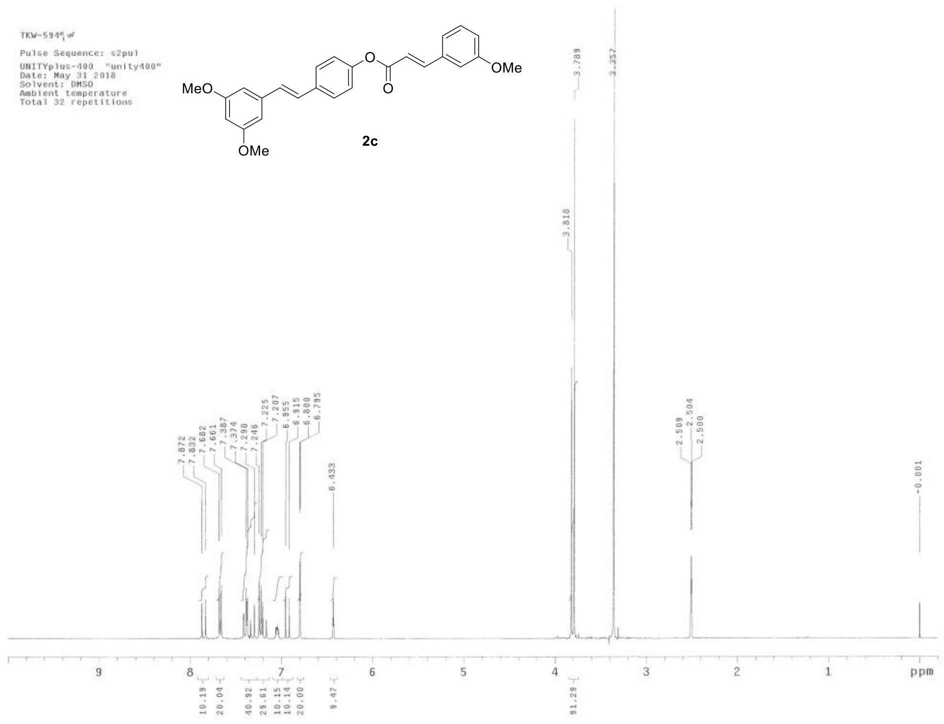
TKW-594
Pulse Sequence: s2pul
UNITYplus-400 "unity400"
Date: May 31 2018
Solvent: DMSO
Ambient temperature
Total 32 repetitions
2c
ppm

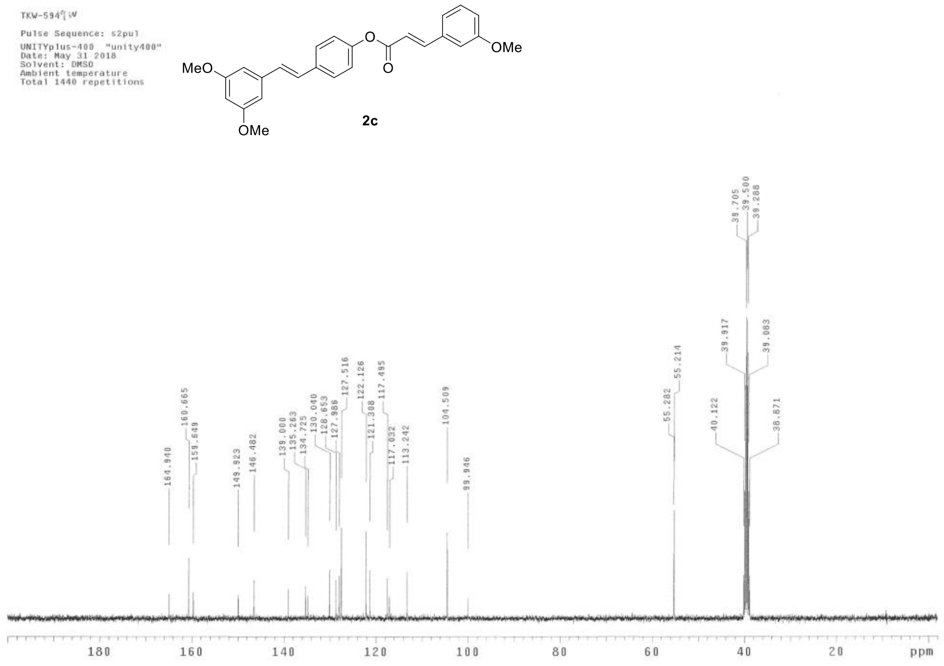
TKW-594
Pulse Sequence: s2pul
UNITYplus-400 "unity400"
Date: May 31 2018
Solvent: DMSO
Ambient temperature
Total 1440 repetitions
2c
ppm

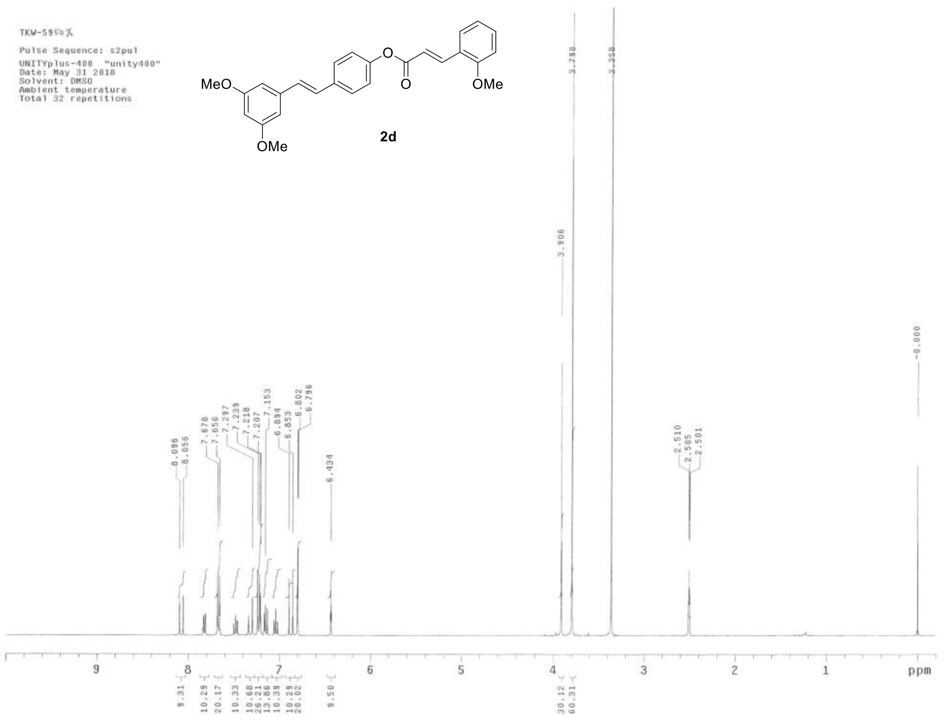
TKW-59
Pulse Sequence: s2pul
UNITYplus-400 "unity400"
Date: May 31 2018
Solvent: DMSO
Ambient temperature
Total 32 repetitions
MeO
OMe
O
O
OMe
2d
ppm

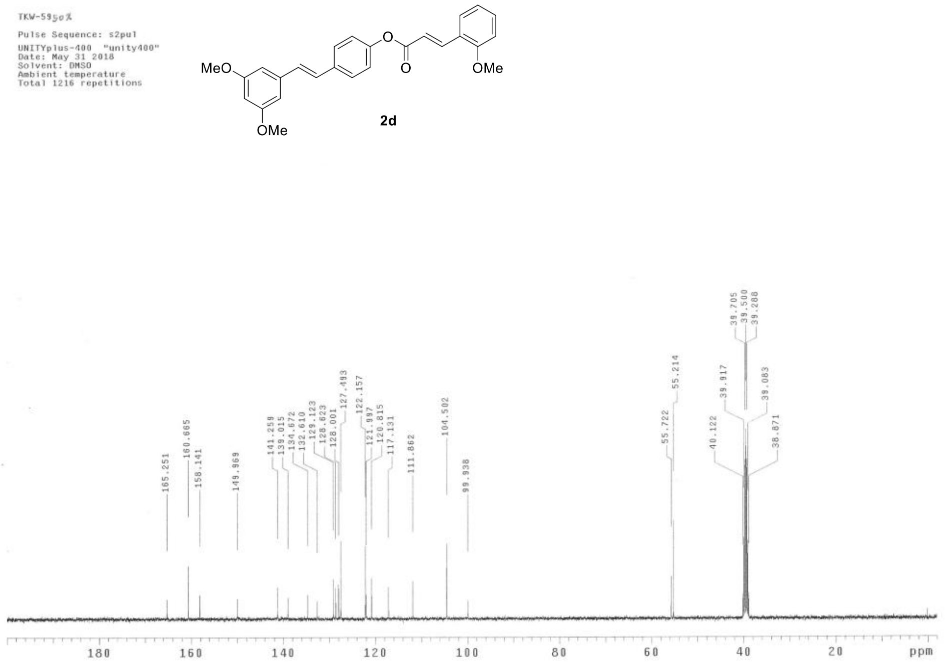
TKW-59
Pulse Sequence: s2pul
UNITYplus-400 "unity400"
Date: May 31 2018
Solvent: DMSO
Ambient temperature
Total 1216 repetitions
MeO
OMe
O
O
OMe
2d
ppm

TKW-6101a
Pulse Sequence: s2pul
UNITYplus-400 "unity400"
Date: Jul 12 2018
Solvent: DMSO
Ambient temperature
Total 32 repetitions

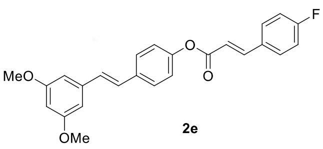

2e

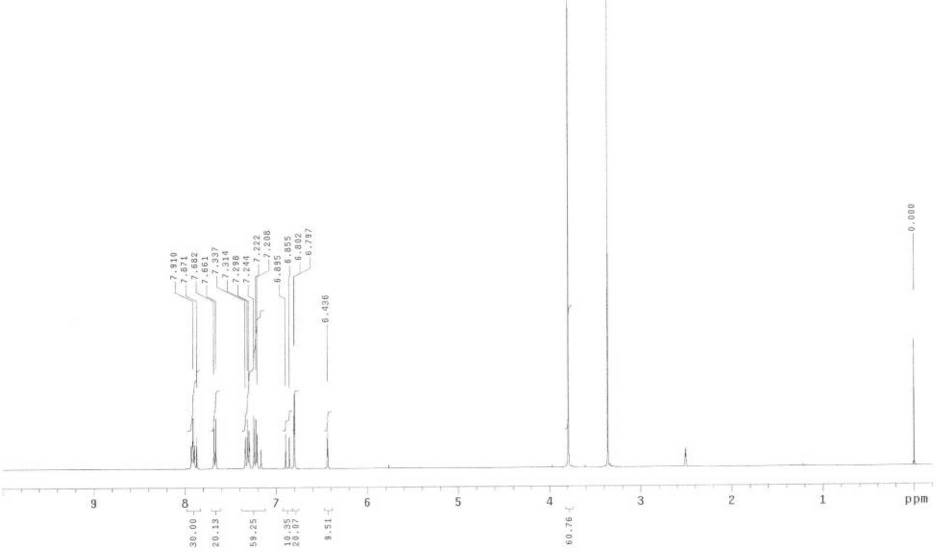

TKW-6101a
Pulse Sequence: s2pul
UNITYplus-400 "unity400"
Date: Jul 12 2018
Solvent: DMSO
Ambient temperature
Total 2720 repetitions

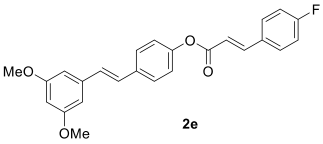

2e

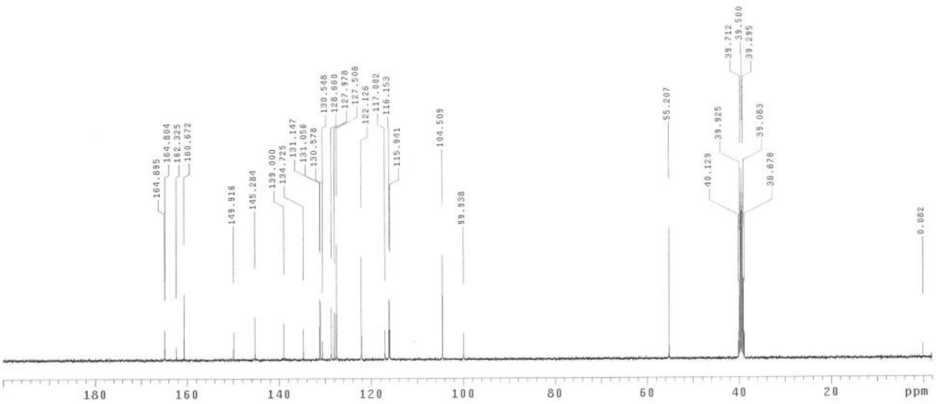

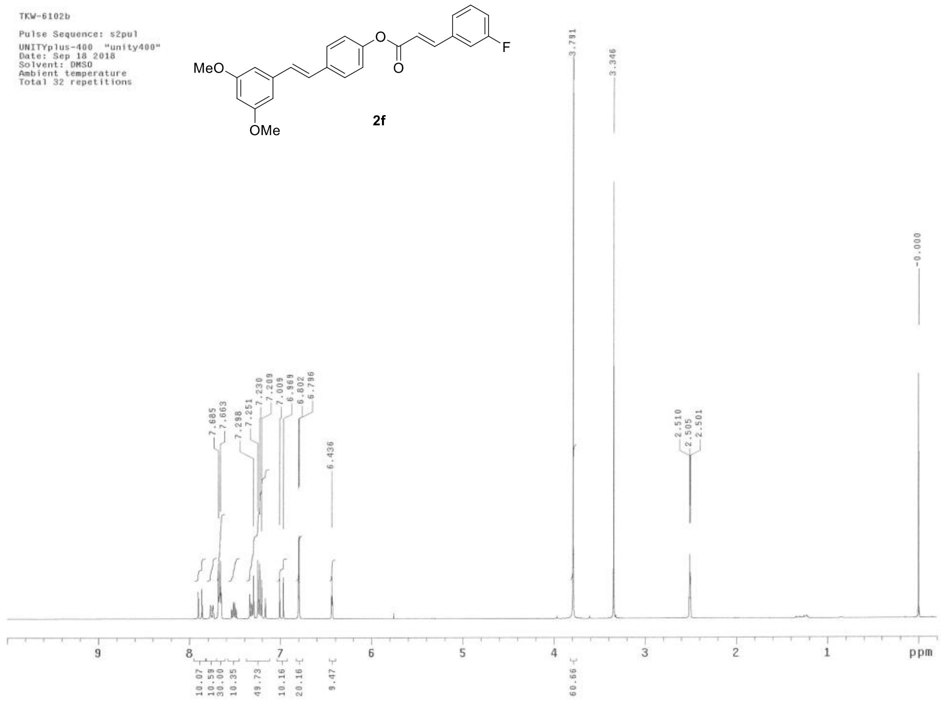
TKW-6102b
Pulse Sequence: s2pul
UNITYplus-400 "unity400"
Date: Sep 18 2018
Solvent: DMSO
Ambient temperature
Total 32 repetitions
MeO
OMe
O
O
F
2f
7.685
7.663
7.298
7.251
7.230
7.209
7.009
6.989
6.802
6.796
6.436
3.791
3.346
2.510
2.505
2.501
0.000
10.07
10.59
30.00
10.35
49.73
10.16
20.16
9.47
60.66
9
8
7
6
5
4
3
2
1
ppm

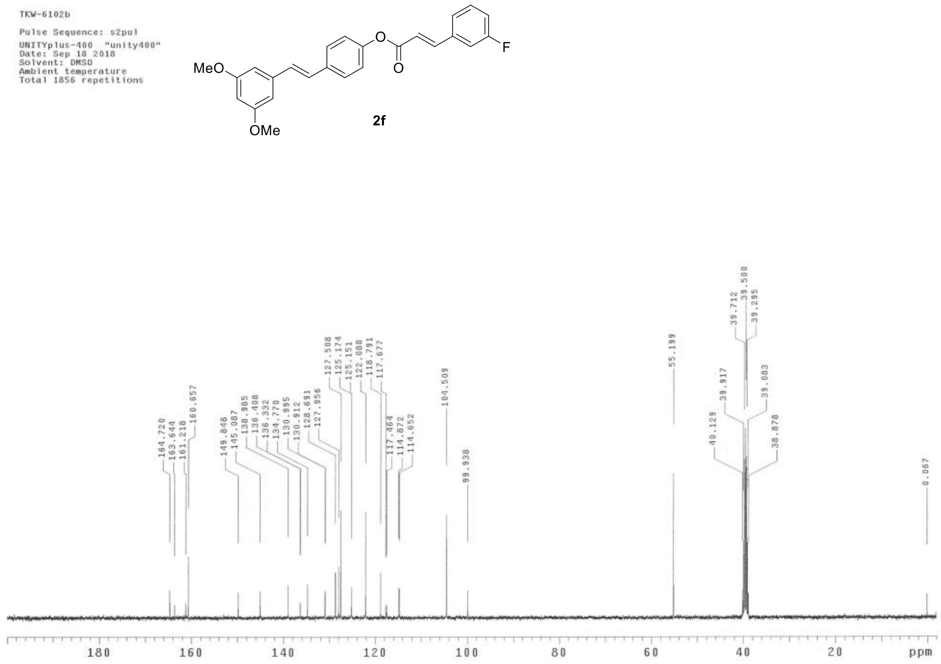
TKW-6102b
Pulse Sequence: s2pul
UNITYplus-400 "unity400"
Date: Sep 18 2018
Solvent: DMSO
Ambient temperature
Total 1856 repetitions
MeO
OMe
O
O
F
2f
164.720
163.644
161.218
160.657
149.848
145.087
138.865
136.408
136.332
134.770
130.995
130.912
128.691
127.956
127.508
125.174
125.151
122.088
118.791
117.677
117.464
114.872
114.652
104.509
99.938
55.199
40.129
39.917
39.712
39.500
39.295
39.083
38.878
0.067
180
160
140
120
100
80
60
40
20
ppm

TKW-6103c
Pulse Sequence: s2pul
UNITYplus-400 "unity400"
Date: Sep 18 2018
Solvent: DMSO
Ambient temperature
Total 32 repetitions

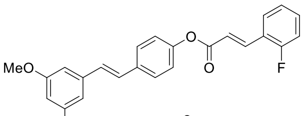

2g

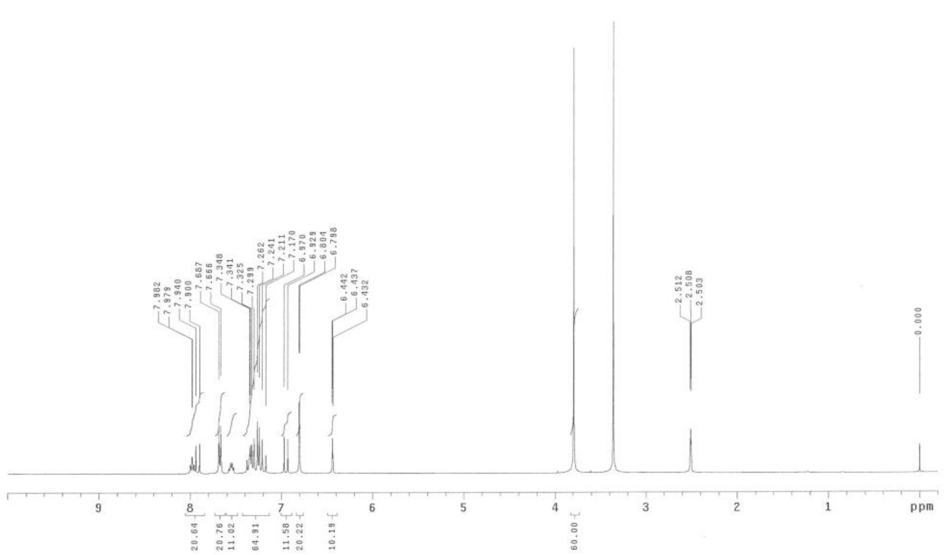

TKW-6103c
Pulse Sequence: s2pul
UNITYplus-400 "unity400"
Date: Sep 18 2018
Solvent: DMSO
Ambient temperature
Total 2304 repetitions

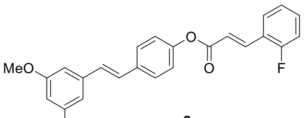

2g

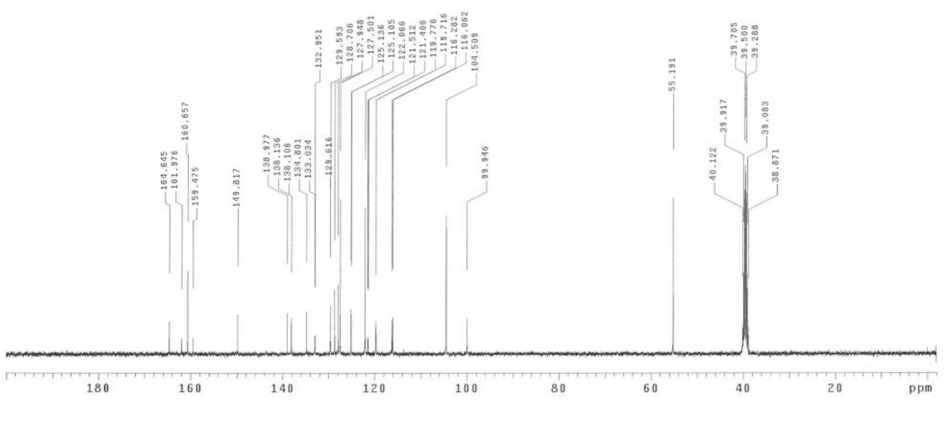

TKW-6104d
Pulse Sequence: s2pul
UNITYplus-400 "unity400"
Date: Sep 18 2018
Solvent: DMSO
Ambient temperature
Total 32 repetitions

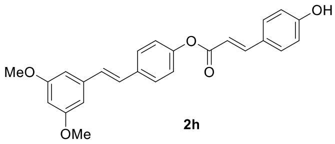

2h

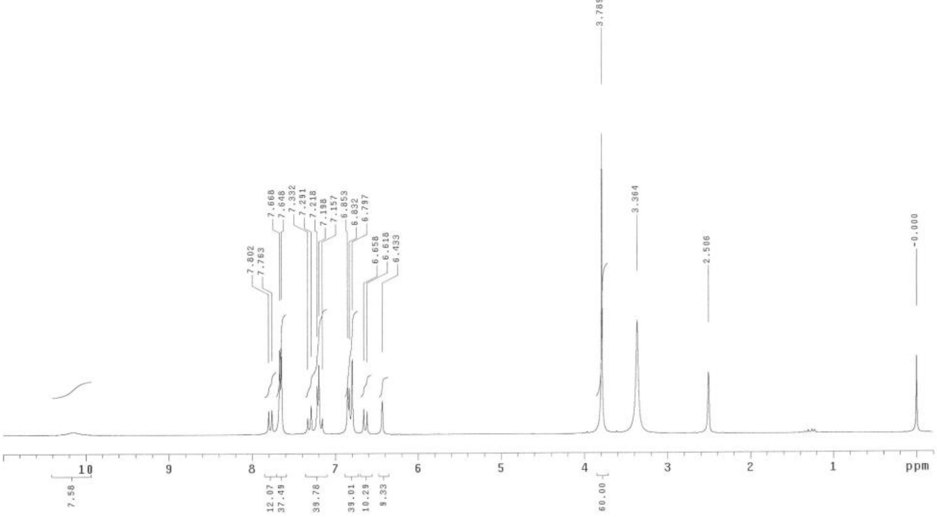

TKW-6104d
Pulse Sequence: s2pul
UNITYplus-400 "unity400"
Date: Sep 18 2018
Solvent: DMSO
Ambient temperature
Total 1264 repetitions

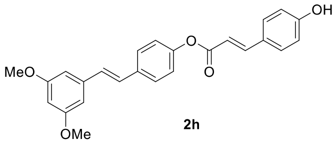

2h

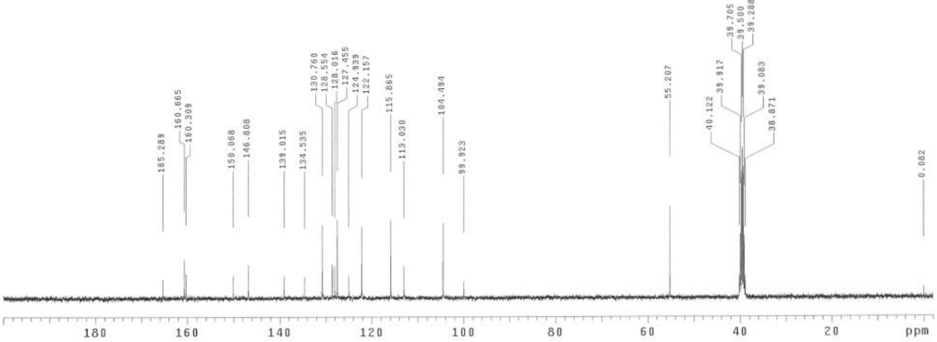

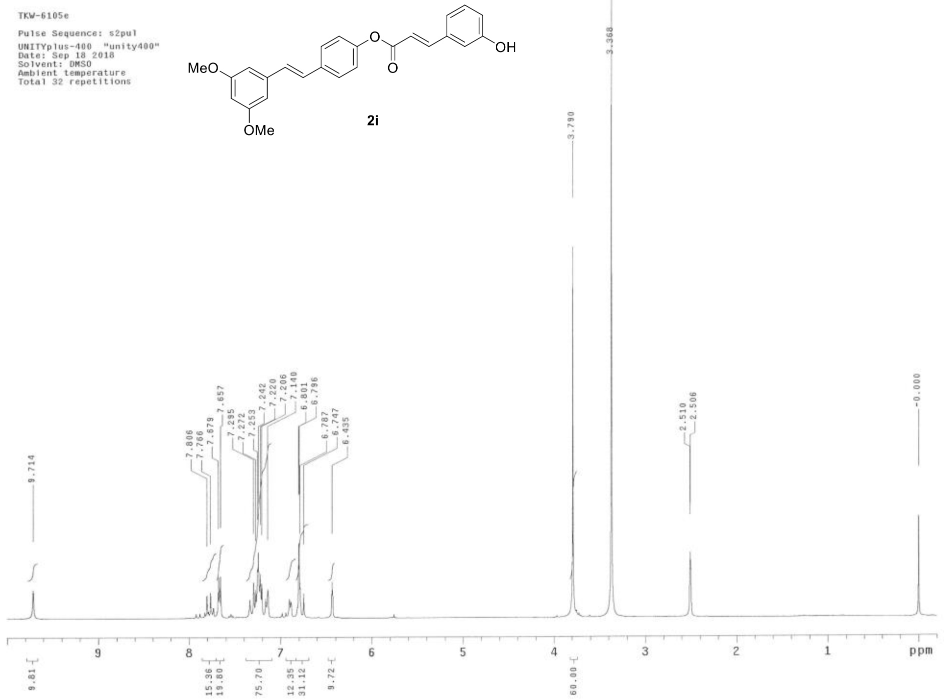
TKW-6105e
Pulse Sequence: s2pul
UNITYplus-400 "unity400"
Date: Sep 18 2018
Solvent: DMSO
Ambient temperature
Total 32 repetitions
MeO
OMe
OH
2i
ppm

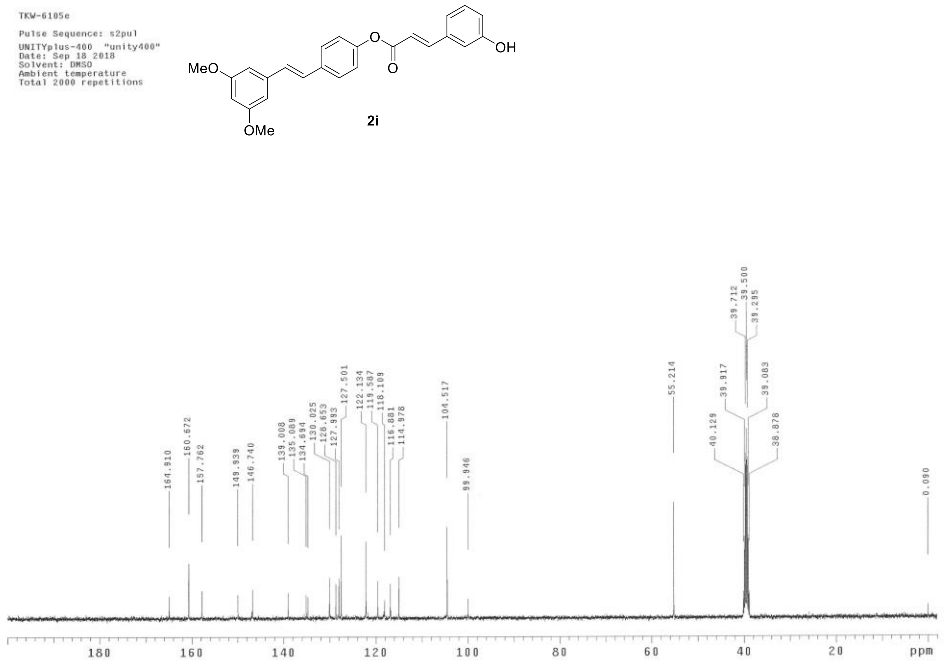
TKW-6105e
Pulse Sequence: s2pul
UNITYplus-400 "unity400"
Date: Sep 18 2018
Solvent: DMSO
Ambient temperature
Total 2000 repetitions
MeO
OMe
OH
2i
ppm

TKW-6106f
Pulse Sequence: s2pul
UNITYplus-400 "unity400"
Date: Jan 9 2019
Solvent: DMSO
Ambient temperature
Total 32 repetitions

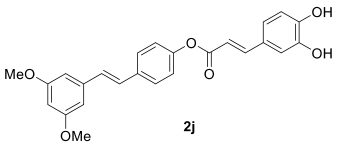

2j

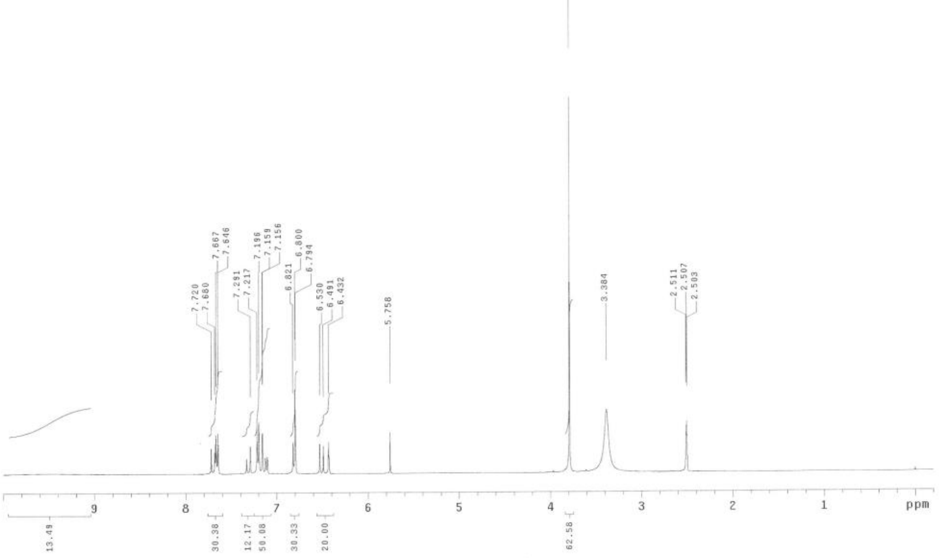

TKW-6106f
Pulse Sequence: s2pul
UNITYplus-400 "unity400"
Date: Jan 9 2019
Solvent: DMSO
Ambient temperature
Total 2384 repetitions

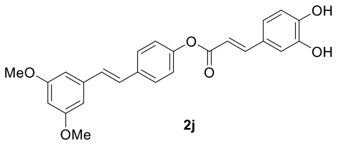

2j

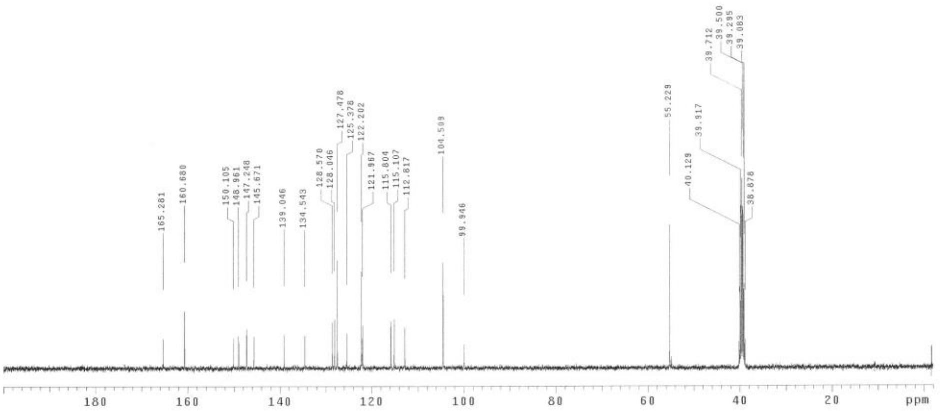

TKW-6157g
Pulse Sequence: s2pul
UNITYplus-400 "unity400"
Date: Jan 9 2019
Solvent: DMSO
Ambient temperature
Total 48 repetitions

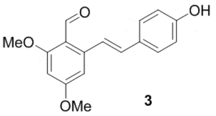

3

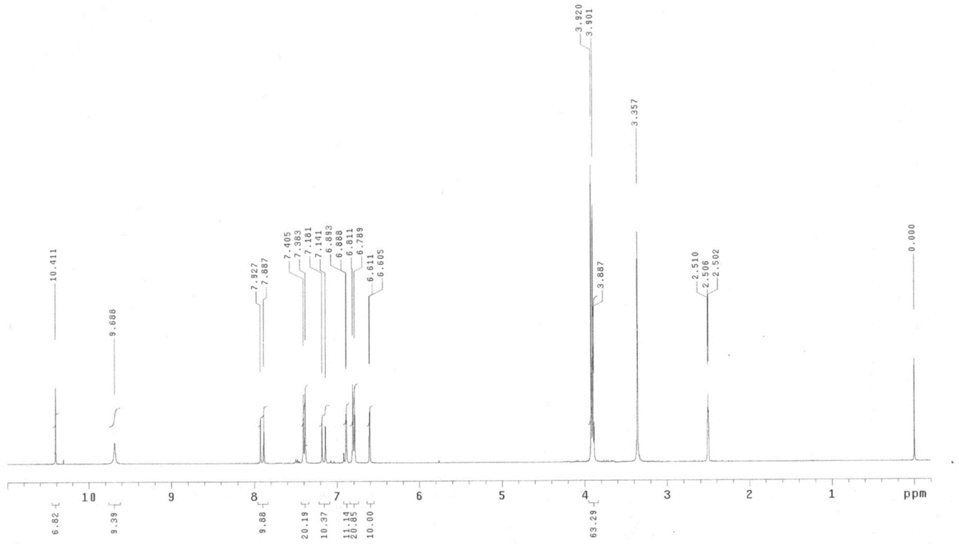

TKW-6157g
Pulse Sequence: s2pul
UNITYplus-400 "unity400"
Date: Jan 9 2019
Solvent: DMSO
Ambient temperature
Total 2048 repetitions

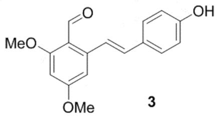

3

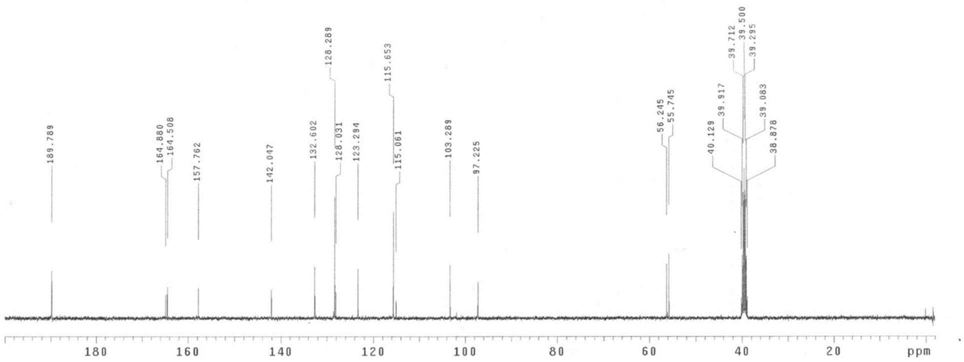

TKW-6159i

Pulse Sequence: s2pul
UNITYplus-400 "unity400"
Date: Mar 18 2019
Solvent: DMSO
Ambient temperature
Total 32 repetitions

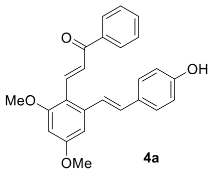

4a

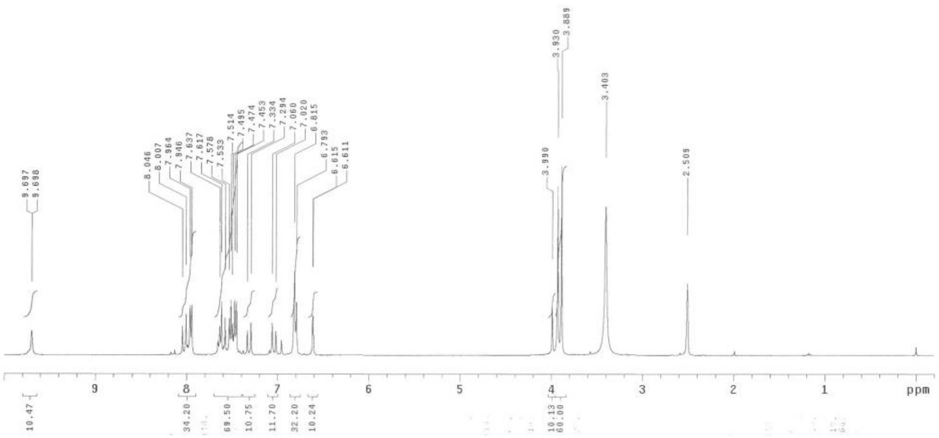

TKW-6159i

Pulse Sequence: s2pul
UNITYplus-400 "unity400"
Date: Mar 18 2019
Solvent: DMSO
Ambient temperature
Total 2704 repetitions

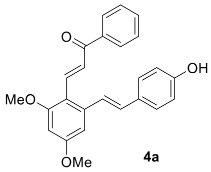

4a

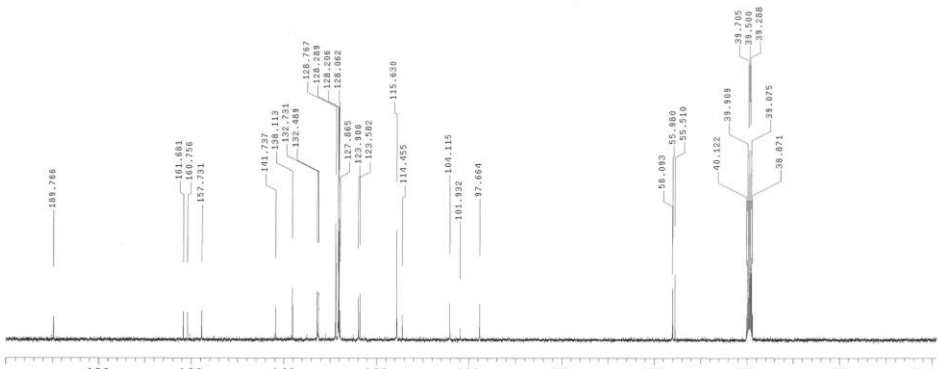

TKW-6158h
Pulse Sequence: s2pul
UNITYplus-400 "unity400"
Date: Feb 21 2019
Solvent: DMSO
Ambient temperature
Total 32 repetitions

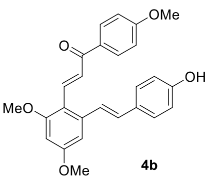

4b

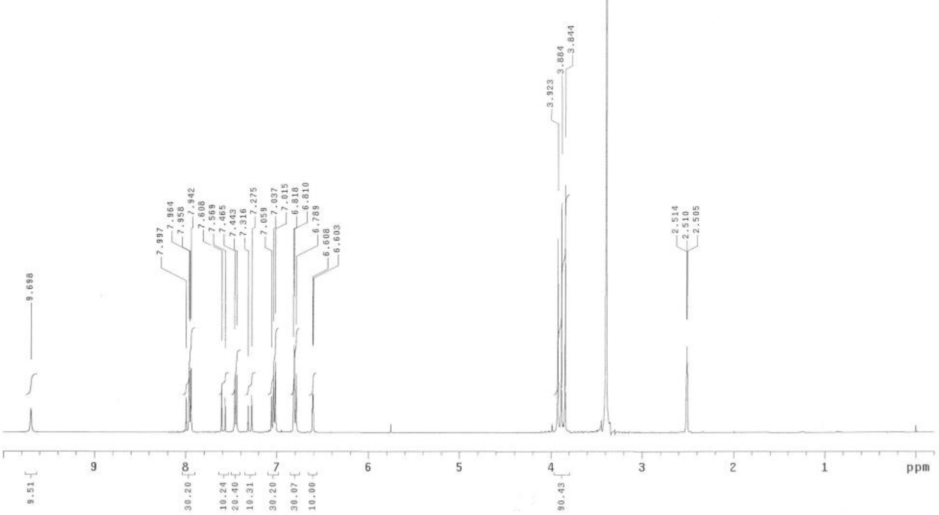

TKW-6158h
Pulse Sequence: s2pul
UNITYplus-400 "unity400"
Date: Feb 21 2019
Solvent: DMSO
Ambient temperature
Total 3008 repetitions

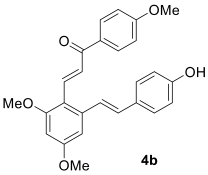

4b

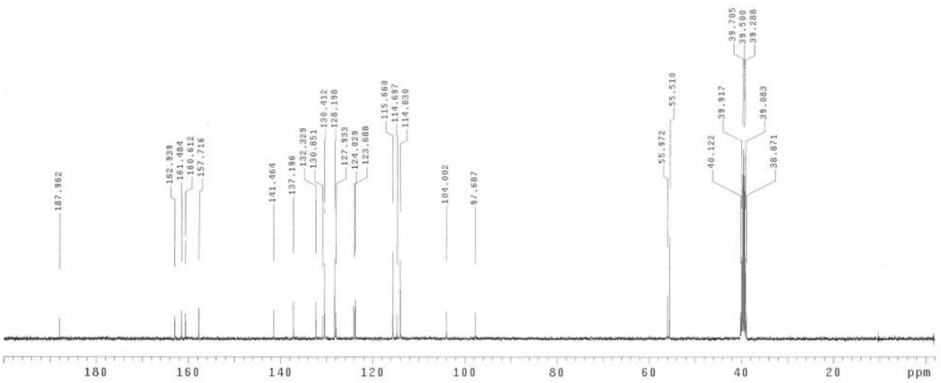

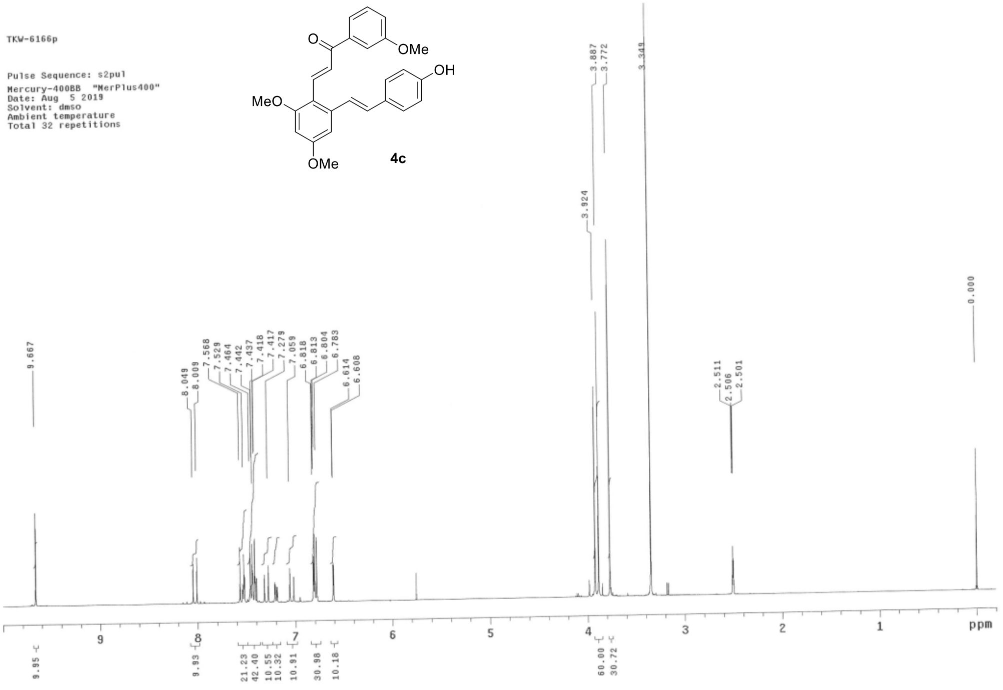
TKW-6166p
Pulse Sequence: s2pul
Mercury-400BB "MerPlus400"
Date: Aug 5 2019
Solvent: dmso
Ambient temperature
Total 32 repetitions
OMe
OH
MeO
OMe
4c
ppm

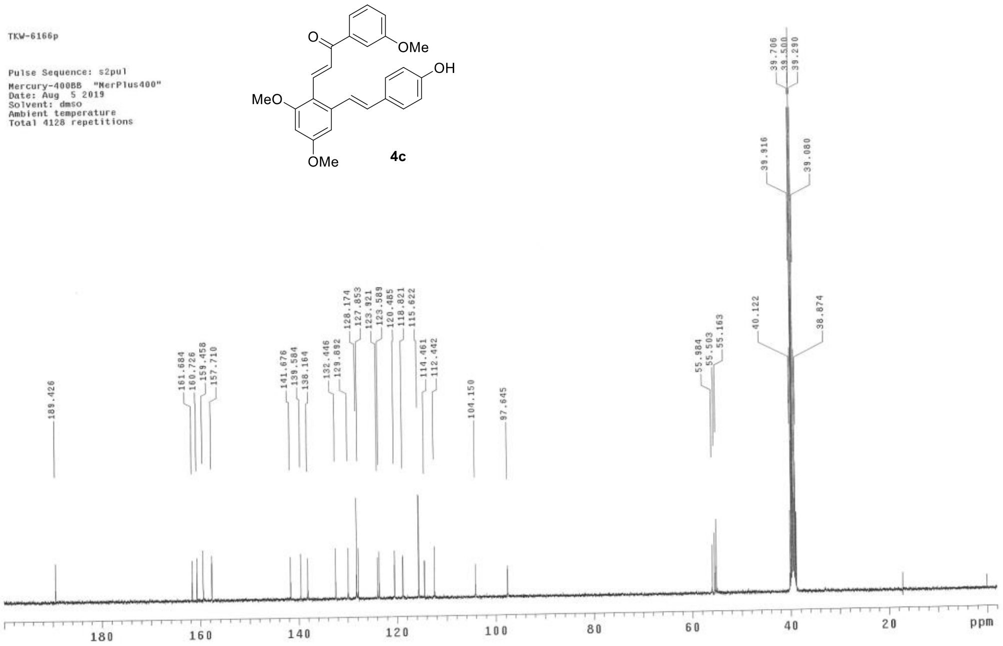
TKW-6166p
Pulse Sequence: s2pul
Mercury-400BB "MerPlus400"
Date: Aug 5 2019
Solvent: dmso
Ambient temperature
Total 4128 repetitions
OMe
OH
MeO
OMe
4c
ppm

TKW-61621
Pulse Sequence: s2pul
UNITYplus-400 "unity400"
Date: Apr 24 2019
Solvent: DMSO
Ambient temperature
Total 32 repetitions

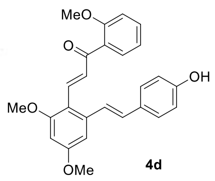

4d

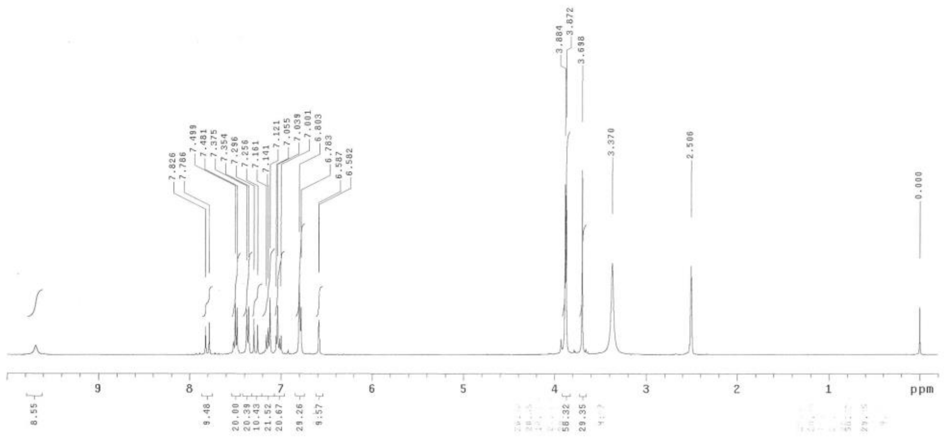

TKW-61621
Pulse Sequence: s2pul
UNITYplus-400 "unity400"
Date: Apr 24 2019
Solvent: DMSO
Ambient temperature
Total 2048 repetitions

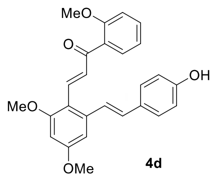

4d

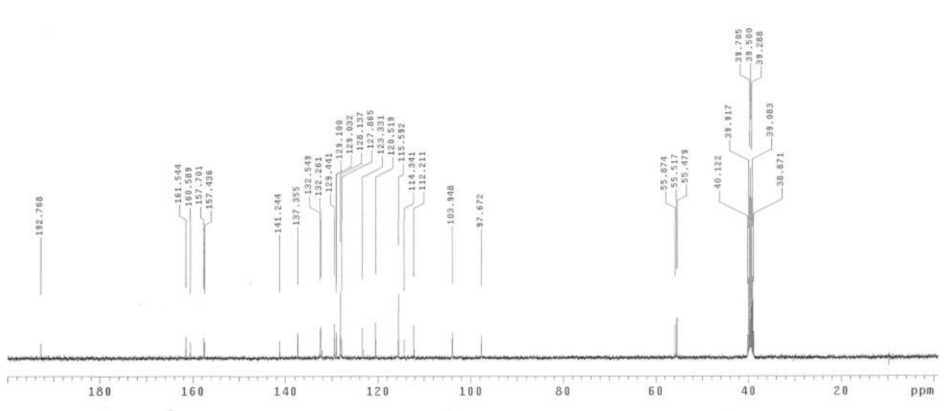

TKW-6160j

Pulse Sequence: s2pul
UNITYplus-400 "unity400"
Date: Mar 18 2019
Solvent: DMSO
Ambient temperature
Total 32 repetitions

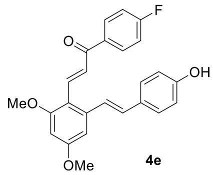

4e

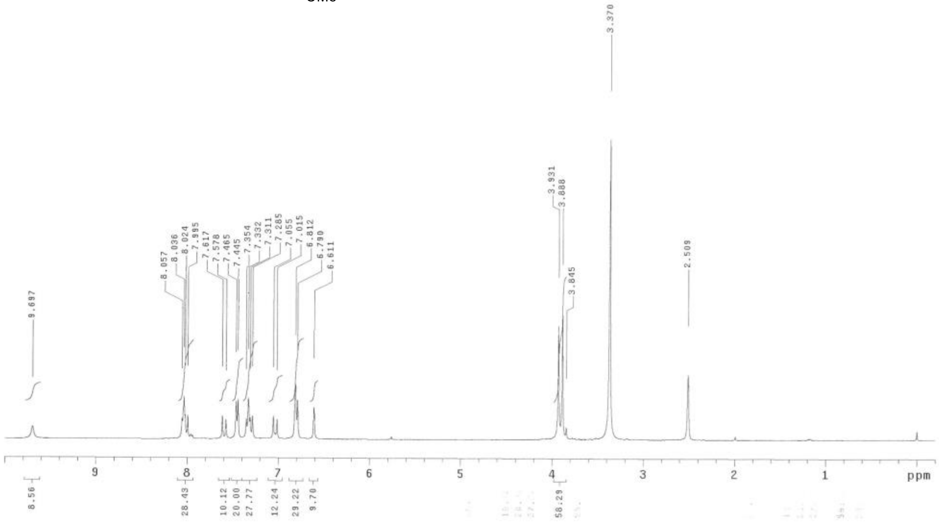

TKW-6160j

Pulse Sequence: s2pul
UNITYplus-400 "unity400"
Date: Mar 18 2019
Solvent: DMSO
Ambient temperature
Total 40176 repetitions

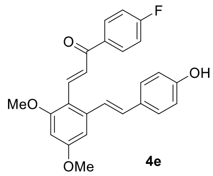

4e

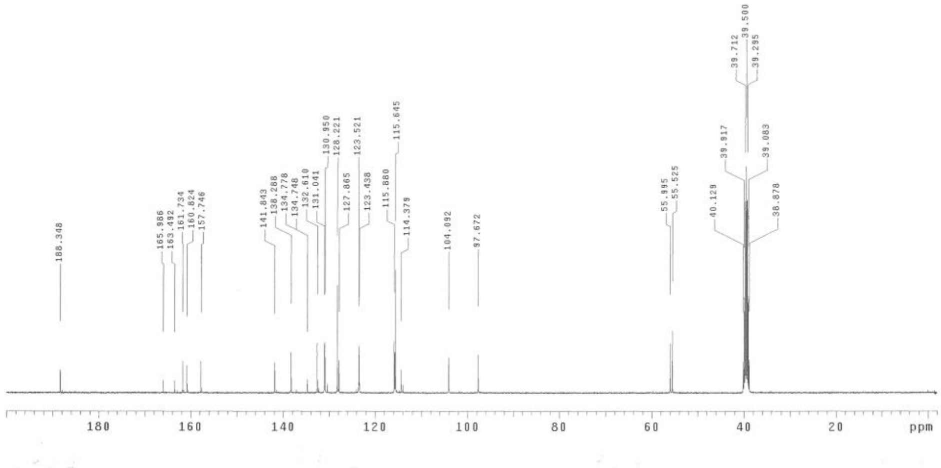

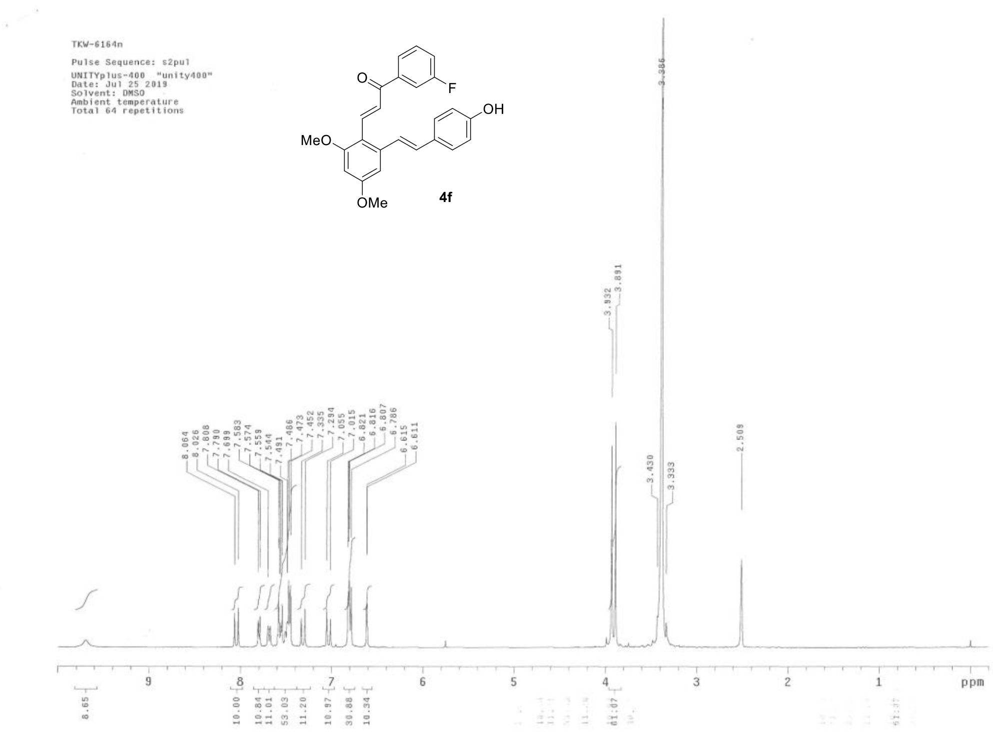
TKW-6164n
Pulse Sequence: s2pul
UNITYplus-400 "unity400"
Date: Jul 25 2019
Solvent: DMSO
Ambient temperature
Total 64 repetitions
MeO
OMe
OH
F
4f
ppm

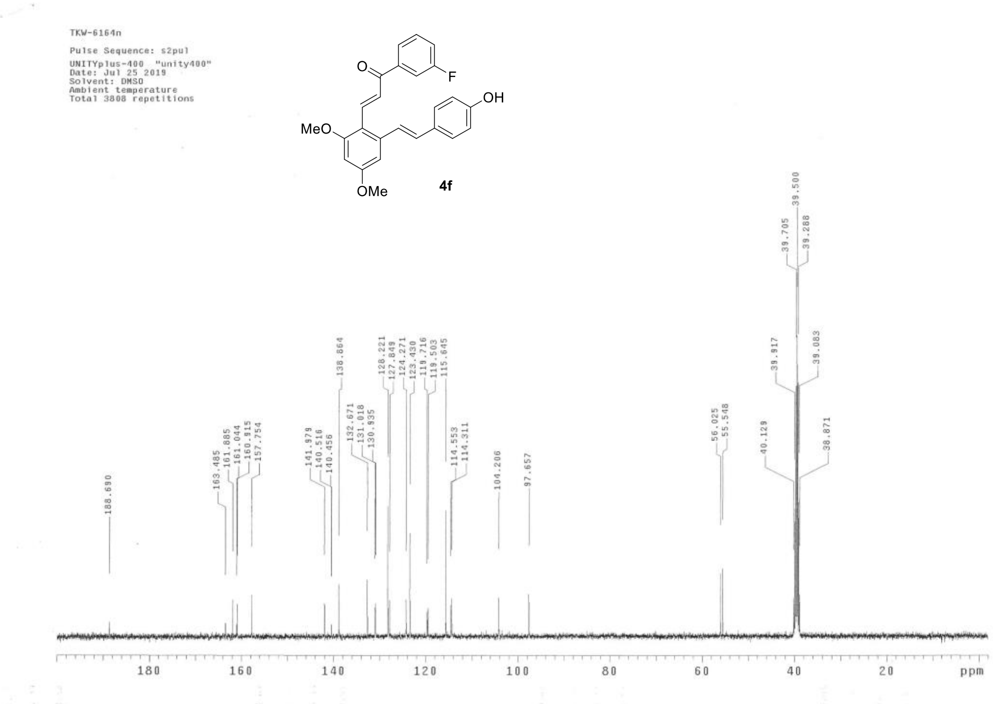
TKW-6164n
Pulse Sequence: s2pul
UNITYplus-400 "unity400"
Date: Jul 25 2019
Solvent: DMSO
Ambient temperature
Total 3808 repetitions
MeO
OMe
OH
F
4f
ppm

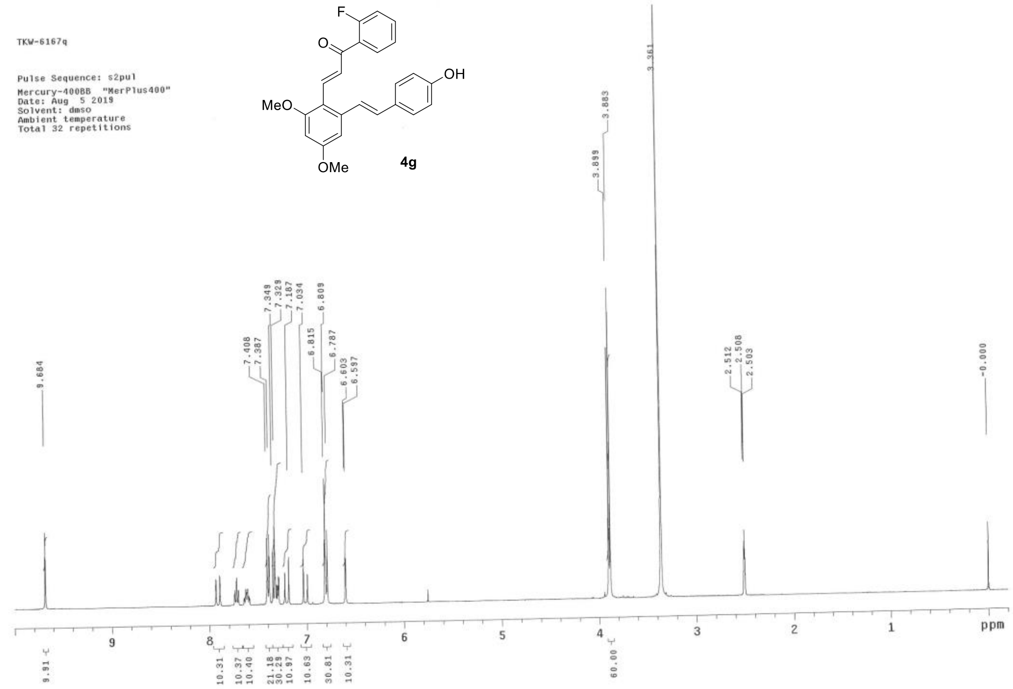
TKW-6167q
Pulse Sequence: s2pul
Mercury-400BB "MerPlus400"
Date: Aug 5 2019
Solvent: dmso
Ambient temperature
Total 32 repetitions
MeO
OMe
OH
4g
ppm

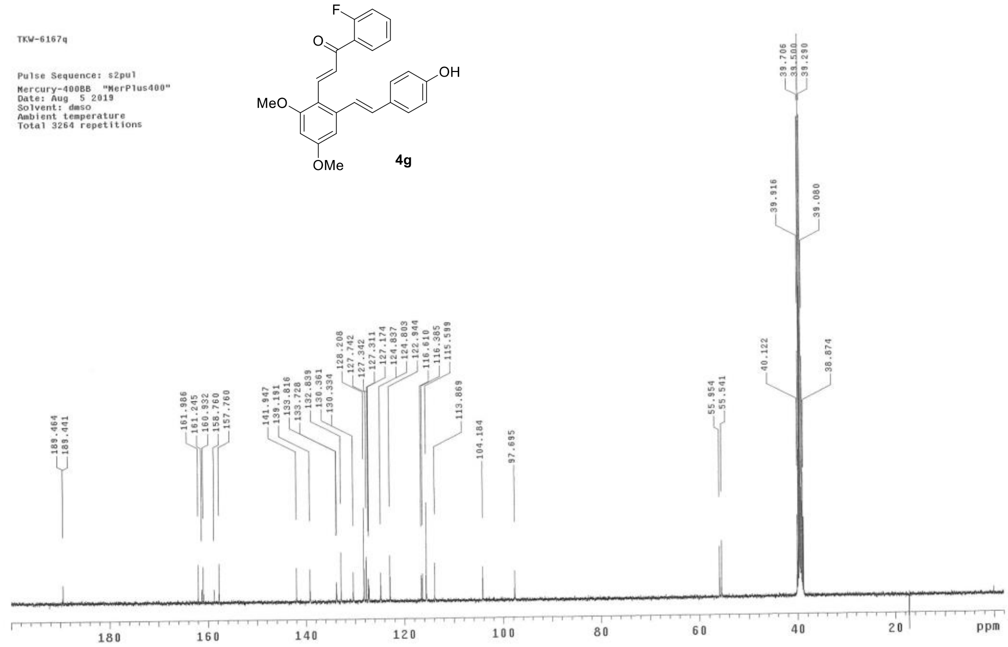
TKW-6167q
Pulse Sequence: s2pul
Mercury-400BB "MerPlus400"
Date: Aug 5 2019
Solvent: dmso
Ambient temperature
Total 3264 repetitions
MeO
OMe
OH
4g
ppm

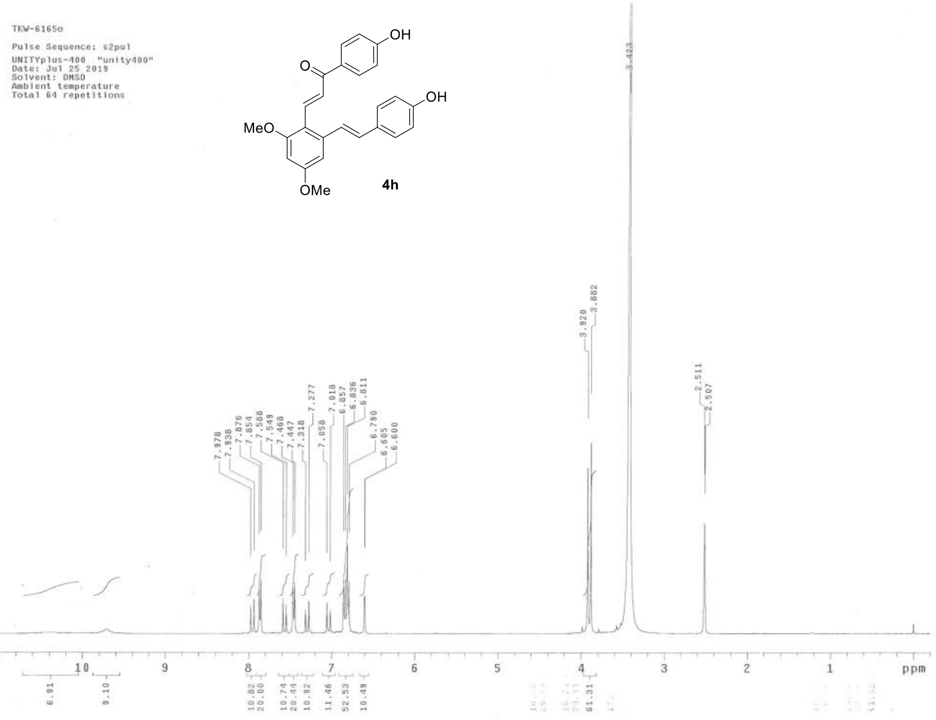
TKW-6165o
Pulse Sequence: s2pul
UNITYplus-400 "unity400"
Date: Jul 25 2019
Solvent: DMSO
Ambient temperature
Total 64 repetitions
OH
O
OH
MeO
OMe
4h
7.978
7.938
7.876
7.854
7.588
7.549
7.468
7.447
7.318
7.277
7.058
7.018
6.857
6.836
6.811
6.790
6.605
6.600
3.920
3.882
3.423
2.511
2.507
10
9
8
7
6
5
4
3
2
1
ppm
6.91
9.10
10.82
20.00
10.74
20.44
10.92
11.46
52.53
10.49
61.31

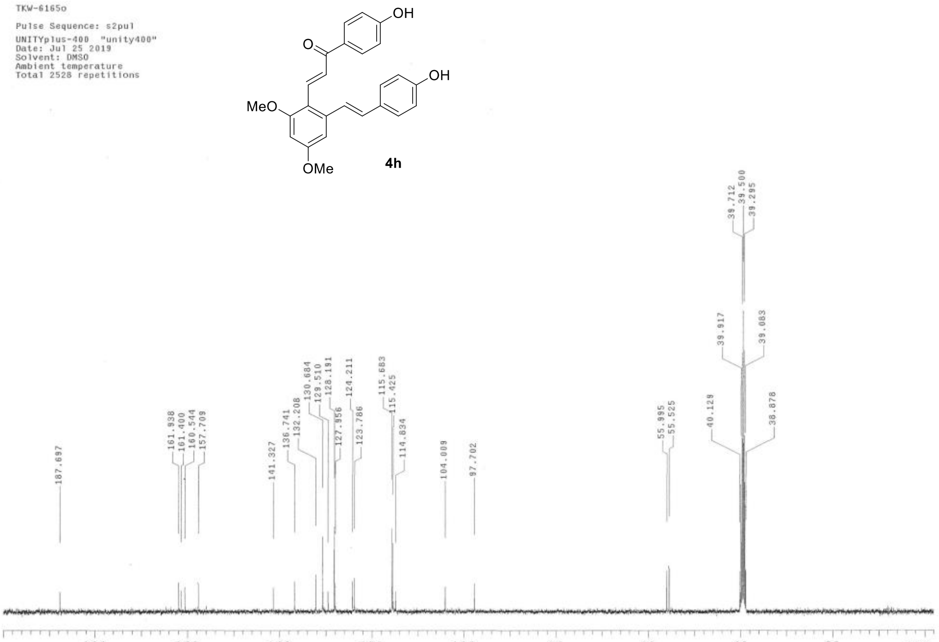
TKW-6165o
Pulse Sequence: s2pul
UNITYplus-400 "unity400"
Date: Jul 25 2019
Solvent: DMSO
Ambient temperature
Total 2528 repetitions
OH
O
OH
MeO
OMe
4h
187.697
161.938
161.400
160.544
157.709
141.327
136.741
132.208
130.684
129.510
128.191
127.956
124.211
123.766
115.683
115.425
114.834
104.089
97.702
55.995
55.525
40.129
39.917
39.712
39.500
39.295
39.083
38.878
180
160
140
120
100
80
60
40
20
ppm

TKW-6163m
Pulse Sequence: s2pul
UNITYplus-400 "unity400"
Date: Apr 24 2019
Solvent: DMSO
Ambient temperature
Total 32 repetitions

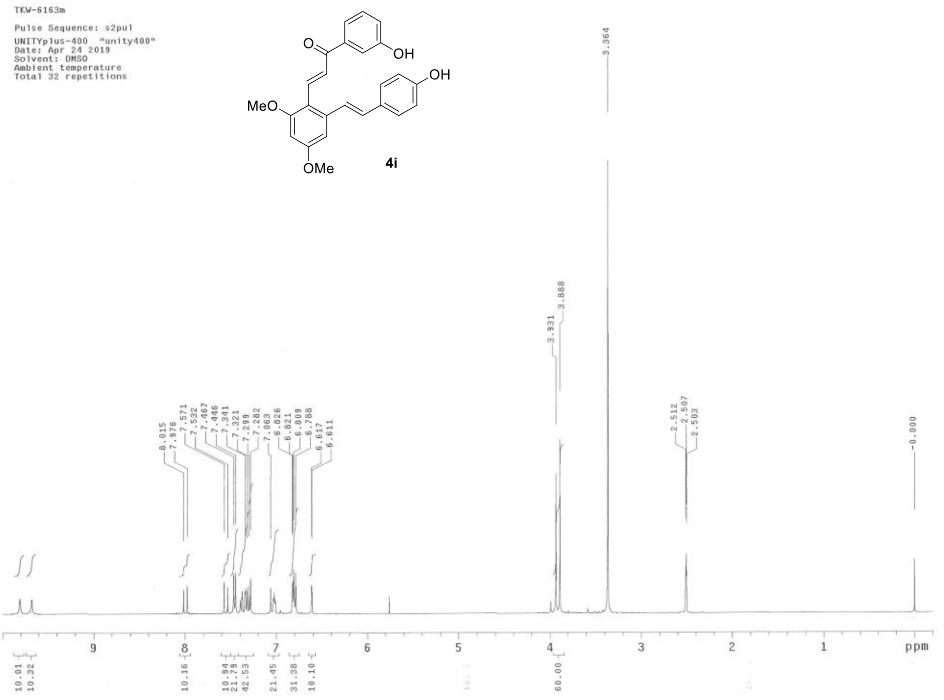

TKW-6163m
Pulse Sequence: s2pul
UNITYplus-400 "unity400"
Date: Apr 24 2019
Solvent: DMSO
Ambient temperature
Total 2736 repetitions

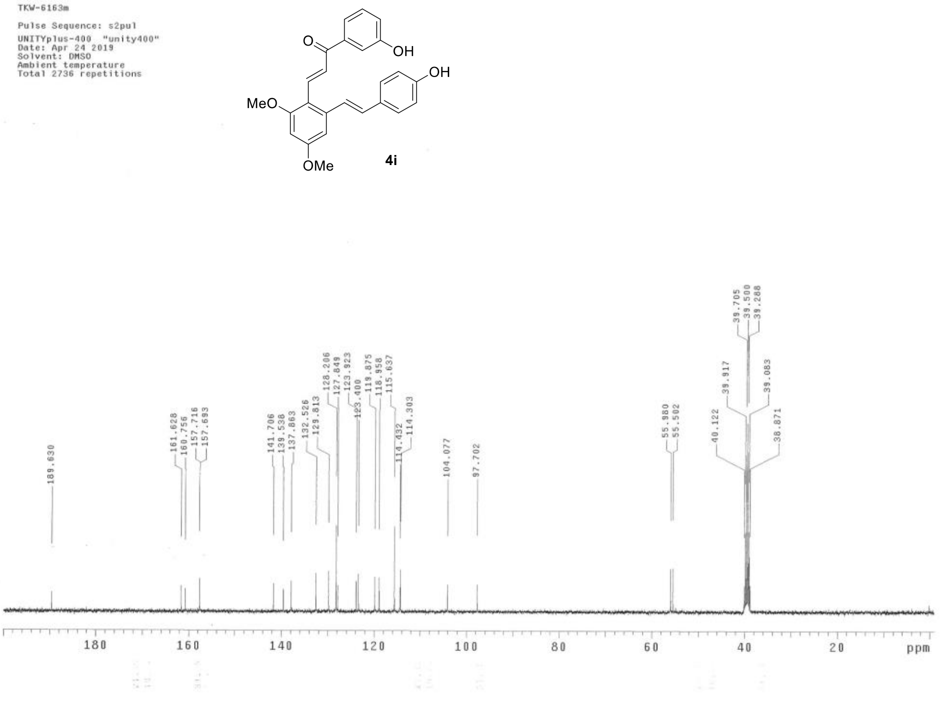

Supplement: Supplementary file 1 [file molecules-26-04840-s001.zip › molecules-1287529-supplementary.pdf]
